# Targeting CXCR2 in Nociceptive Sensory Neurons Offers Novel Therapeutic Potentials for Postoperative Pain

**DOI:** 10.1002/advs.77085

**Published:** 2026-08-11

**Authors:** Yushuang Pan, Peiyi Li, Yufeng Chen, Zishan Dong, Yuanyuan Li, Boyu Liu, Yan Tai, Chuan Wang, Jianqiao Fang, Chengyu Yin, Boyi Liu

**Affiliations:** ^1^ Department of Neurobiology and Acupuncture Research The Third Clinical Medical College Key Laboratory of Acupuncture and Neurology of Zhejiang Province Zhejiang Chinese Medical University Hangzhou China; ^2^ Department of Pathophysiology Hebei Key Laboratory of Critical Disease Mechanism and Intervention Hebei Medical University Shijiazhuang China; ^3^ Hangzhou TCM Hospital Affiliated to Zhejiang Chinese Medical University Hangzhou China; ^4^ Academy of Chinese Medical Sciences Zhejiang Chinese Medical University Hangzhou China; ^5^ College of Pharmaceutical Sciences Hebei University Baoding China; ^6^ Department of Integrated Traditional Chinese and Western Medicine Union Hospital Tongji Medical College Huazhong University of Science and Technology Wuhan China

**Keywords:** CXCR2, dorsal root ganglia, postoperative pain, TRPA1, TRPV1

## Abstract

Postoperative pain impairs patients’ quality of life. Mechanisms underlying postoperative pain remain incompletely understood. We investigated postoperative pain in a mouse model of skin plus deep tissue incision (INC). We found *Cxcl5* was among the top upregulated genes in incision site, which was produced from both incised skin and muscle. Single‐cell RNA‐sequencing reveals elevated human *CXCL5* gene expression in human skin wounds. Neutralizing CXCL5 abrogated INC pain. Global knockout of CXCL5 receptor CXCR2 improved INC pain, but markedly delayed wound healing. *Cxcr2* conditional knockout in nociceptive sensory neurons improved INC pain without affecting wound healing and local inflammation. CXCR2 expression and its coupling with TRPA1 were enhanced in DRG neurons innervating the incised site, resulting in neuron hyperexcitability upon CXCL5 stimulation. CXCL5 further enhances TRPV1 activity via neuronal CXCR2‐mediated signaling in DRG neurons innervating the incised site. Neuronal CXCR2‐mediated TRPV1/TRPA1 modulation synergistically contributes to heat and mechanical hypersensitivities of INC pain. Targeted *Cxcr2* knockdown in incision site‐innervating DRG neurons ameliorates INC pain. Our work reveals a critical role of neuronal CXCR2 signaling in nociceptive sensory neurons that mediates INC pain via concurrently activating TRPA1 and sensitizing TRPV1. Targeting sensory neuronal CXCR2 represents a promising strategy for INC pain without affecting wound healing.

## Graphical abstract

Tissue incision induces CXCL5 release, which activates neuronal CXCR2 on incision‐innervating sensory neurons. Neuronal CXCR2 couples with TRPA1 to drive mechanical pain and engages p38 MAPK signaling to upregulate TRPV1 and promote heat pain. Targeted Cxcr2 knockdown relieves incisional pain while preserving CXCR2‐dependent neutrophil responses required for inflammation and healing.

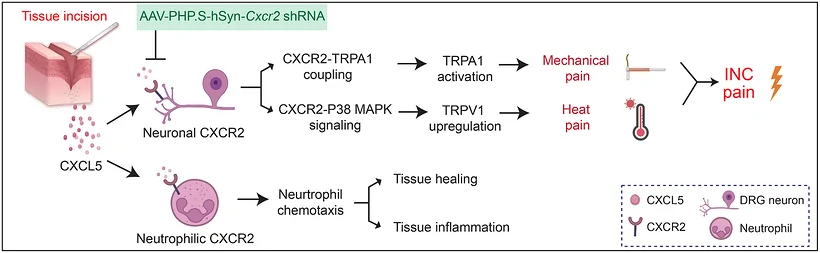

## 1 Introduction

Postoperative pain is a prevalent and distressful condition among patients after surgical procedures [1, 2]. If inadequately managed, postoperative pain severely compromises patients’ quality of life, impedes physical recovery, and exacerbates psychological burdens [2]. Currently, postoperative pain management primarily relies on opioids and nonsteroidal anti‐inflammatory drugs [2, 3]. But a substantial proportion of patients still experience moderate to severe postoperative pain due to the limited efficacy and notable side effects of these treatments. These challenges highlight an urgent need to identify novel and safer targets for more effective postoperative pain treatment.

The incised tissues release a plethora of inflammatory mediators, cytokines, and chemokines [4]. These substances can act on their receptors expressed by nociceptors at nerve terminals of sensory neurons, thereby promoting neuronal hyperexcitability and contributing to incisional pain [4, 5]. While blocking the signaling pathways mediated by these substances may offer pain relief for tissue incision, it is important to note that they also play crucial roles in immune cell recruitment, including macrophages and neutrophils [6, 7]. This process is a critical step for tissue healing and defense against pathogens following tissue incision [7]. Therefore, systematic or untargeted blockade of these substances, their receptors or the associated downstream pathways may impair tissue healing or compromise host defense, thereby posing adverse effects on incisional pain management.

As a member of CXC chemokine family, CXCL5 functions dually as an inflammatory mediator and a powerful neutrophil chemoattractant, with its biological activities largely dependent on interaction with receptor CXCR2 [8]. While CXCR2 is best known for its critical role in neutrophil chemotaxis due to high expression on these immune cells, emerging evidence has revealed its presence in peripheral sensory neurons and spinal cord [9]. It has been shown that CXCR2 expressed on peripheral sensory neurons or spinal cord neurons contributes to pain mechanisms in inflammatory pain, bone cancer pain, and paclitaxel‐induced peripheral neuropathy [10, 11, 12, 13, 14]. This extra‐immune expression profile underscores CXCR2's previously underappreciated contribution to pain modulation.

Here, we identified *Cxcl5* was among the top upregulated genes in incised tissues and neutralizing CXCL5 abrogated pain in a mouse model of skin plus deep tissue incision (INC). Global *Cxcr2* knockout improved INC pain, but caused delay in wound healing, whereas *Cxcr2* conditional knockout in nociceptive sensory neurons improved INC pain without affecting wound healing. CXCR2 expression and its coupling with TRPA1 were enhanced in DRG neurons innervating the incised site, resulting in hyperexcitability of DRG neurons upon CXCL5 activation. CXCL5 also potentiates TRPV1 via neuronal CXCR2 in DRG neurons. These two effects concurrently contributes to INC pain. Moreover, targeted *Cxcr2* knockdown in incision site‐innervating DRG neurons effectively reduced INC pain without affecting tissue healing. Our work reveals a critical role of neuronal CXCR2 signaling in nociceptive sensory neurons mediating INC pain. Targeting sensory neuronal CXCR2 may represent an effective strategy for INC pain relief without affecting wound healing process.

## 2 Materials and Methods

### 2.1 Animals

C57BL/6 male or female mice (6–8 weeks) were purchased from Shanghai Laboratory Animal Center. SNS‐Cre mice on a C57BL/6 background were provided by Professor Chuan Wang. To conditionally delete *Cxcr2* in nociceptors, we crossed *Cxcr2* floxed (*Cxcr2* ^fl/fl^) mice with SNS‐Cre mice to produce SNS‐Cre *Cxcr2* ^fl/fl^ offspring, hereafter referred to as *Cxcr2* cKO. A global *Cxcr2* knockout (*Cxcr2* gKO) strain was obtained from GemPharmatech (Nanjing, China). The mice were housed in group of 5 per cage within a specific pathogen‐free facility under standard conditions (12‐h light/dark cycle, ambient temperature of 24°C ± 2°C, and relative humidity of 50%–60%). Upon arrival, animals were acclimatized for 7 days before test. Cage assignment was randomized. Experimental procedures were approved by the Animal Ethics Committee of Zhejiang Chinese Medical University (#IACUC‐20201214‐13).

### 2.2 Skin Plus Deep Tissue Incision Model Establishment

The model was adopted from previous studies [6, 15]. Surgery was performed under anesthesia induced with isoflurane. A 5‐mm longitudinal incision was made through the skin and underlying fascia with a sterile No. 11 scalpel blade, beginning 2 mm from the proximal edge of the heel. To incorporate a deep tissue injury, the flexor digitorum brevis muscle was gently elevated from its bed using curved forceps while leaving its origin and insertion intact. The skin incision was then apposed with a single 6‐0 nylon mattress suture. The suture was removed on the third post‐operative day. Sham‐operated controls received identical anesthetic and sterile preparation but did not undergo incision or muscle manipulation.

### 2.3 Skin Wound Creation and Evaluation

A standardized full‐thickness excisional wound model was employed as previously described [16]. Briefly, mice were anesthetized via intraperitoneal injection of sodium pentobarbital. After shaving the dorsal hair, a full‐thickness excisional wound was created on each mouse's dorsum using a dermal biopsy punch. Following the procedure, animals recovered on a circulating water heating pad until fully ambulatory. Wound closure was monitored through digital photography and subsequent image analysis. On post‐wounding days 1, 5, and 7, mice were re‐anesthetized, their wounds were gently cleaned, and any regrown hair was removed. A standardized reference scale was placed adjacent to the wounds for imaging from a fixed distance of 20 cm. The area of each open wound was quantified using ImageJ. The wound area on day 1 served as the baseline, representing 100% wounding area of initial area. The percentage of wound closure at later time points was calculated by comparing the remaining open wound area to this baseline value.

### 2.4 Mechanical Allodynia

Mice were placed individually in a transparent plastic chamber set on an elevated wire‐mesh platform and covered with a transparent acrylic lid to allow habituation for approximately 30 min. Mechanical sensitivity was assessed as described before [17]. After a mouse was fully quiet and settled, von Frey filaments were applied to the central plantar surface of ipsilateral hind paw. Each filament was pressed perpendicularly until it bent into an S‐shape, held in place for approximately 5 s, and the presence or absence of a paw‐withdrawal reflex was recorded. The up‐down testing paradigm was used to determine the paw withdrawal threshold (PWT) as previously described [18, 19]. The PWT was calculated using nonparametric Dixon method [20]. Paw withdrawal frequencies in response to mechanical stimuli were tested as described before [21]. The mouse was placed individually in a transparent plastic chamber on an elevated mesh. Two von Frey filaments (0.07 and 0.4 g) were applied to the hind paw for approximately 1 s, respectively. Each stimulation was repeated ten times to the hind paw. The occurrence of paw withdrawal in each of these ten trials was expressed as a percent response frequency ((number of paw withdrawals/10 trials) × 100 = % response frequency).

### 2.5 Heat Hyperalgesia

Heat hyperalgesia was assessed by the Hargreaves method as described before [22]. Mice were placed individually in a transparent acrylic observation chamber positioned on a glass plate (2 mm thickness), with the plantar surface of the hindpaw exposed to a radiant heat stimulus generated by a Hargreaves apparatus (Ugo, Italy). To prevent tissue injury, a cut‐off time of 20 s was imposed, and the inter‐trial interval was set to 5 min. Each animal underwent three trials; the paw withdrawal latency (PWL) was recorded for each trial and the arithmetic mean of the three measurements was used for statistical analysis.

### 2.6 Gait Analysis

Gait analysis was evaluated using DigiGait imaging system (Mouse Specifics, Inc., USA) as described before [23]. Animals were habituated to the apparatus for 3 days prior to testing. During assessment, mice were allowed to freely traverse a walkway of predefined speed (18 cm/s), while a high‐speed camera recorded their paw placements. Paw prints were captured using an internal light‐based paw‐print illumination/reflection technique and the recorded videos were processed with the manufacturer's software to obtain gait parameters. All measurements were performed under darkroom conditions.

### 2.7 Elevated Plus Maze (EPM) Test

Mice were acclimated in a temperature‐controlled room (25°C) with the lights turned off for 30 min. The EPM was disinfected with 75% ethanol and then rinsed with water to remove residual ethanol odor before the first trial. Each mouse was placed in the center of the EPM (6 × 6 cm) and allowed a 30 s habituation period, after which the experimenter left the room. Behavioral tracking was performed using ANY‐maze software (Stoelting Co., USA) to record total distance traveled and the time spent in the open arms (30 × 6 cm).

### 2.8 Open Field Test (OFT)

Mice were acclimated in a temperature‐controlled room (25°C) with the lights off for 30 min. The OFT box (40 × 40 cm) was disinfected with 75% ethanol and then rinsed with water to remove residual ethanol odor. Each mouse was placed in the center area of the arena (20 × 20 cm) and allowed a 30 s habituation period. The behavioral tracking was then initiated using ANY‐maze software (Stoelting Co., USA) to record total distance traveled and time spent exploring the center area.

### 2.9 ELISA

Skin or muscle samples were homogenized in RIPA buffer. Homogenates were centrifuged at 12 000 × g for 12 min at 4°C. Protein concentrations in the supernatants were measured using a BCA assay (Thermo Fisher Scientific, USA). CXCL5 protein levels were quantified in duplicates by ELISA using a commercial kit (Elabscience Biotechnology, China) following the manufacturer's protocol.

### 2.10 qPCR

Ipsilateral hindpaw skin or muscle samples were collected from mice and total RNA was extracted using an RNA isolation kit (TaKaRa, China). cDNA was synthesized from 1 µg of total RNA using the PrimeScript RT Reagent Kit (TaKaRa, China). qPCR was performed on a CFX96 Real‐Time System (Bio‐Rad Laboratories, USA) with a 20 µL reaction volume. Relative gene expression was determined by the comparative cycle threshold (^ΔΔ^Ct) method [24, 25]. β‐actin (*Actb*) was used as internal control. All reactions were run in triplicate; Ct values were obtained with the CFX96 software and the mean of the triplicates was used for analysis. Primer sequences (5'→3') for Actb were as follows: *Actb* forward, 5'‐GTGCTATGTTGCTCTAGACTTCG‐3'; *Actb* reverse, 5'‐ATGCCACAGGATTCCATACC‐3'. *Cxcl5* forward, 5'‐TGATCGCTAATTTGGAG GTGAT‐3'; *Cxcl5* reverse, 5'‐TAGCTTTCTTTTTGTCA‐3'.

### 2.11 DRG Neuron Culture

DRG neurons were isolated and cultured following an established protocol with minor modifications [26]. Briefly, DRG are dissected and digested for 1 h at 37°C using a DMEM solution containing 1 mg/ml collagenase A and 2 mg/mL dispase (Gibco, Thermo Fisher Scientific, USA). After trituration and centrifugation, the isolated neurons are plated on coverslips or in eight‐well chambered coverglass (Nunc, Denmark) pre‐coated with poly‐D‐lysine (Sigma, USA) and mouse laminin (Thermo Fisher Scientific, USA). The neurons are cultured in DMEM supplemented with 10% FBS (Hyclone, USA), 100 U/mL penicillin, and 100 µg/mL streptomycin.

### 2.12 Whole‐Cell Patch‐Clamp Recording

Whole‐cell patch‐clamp recordings were performed in dissociated DRG neurons with Axopatch 700B amplifier and Digidata 1550B digitizer (Molecular Devices, USA) 6 h after plating as described before [27]. Patch pipettes (3–5 MΩ) were pulled from borosilicate glass capillary tubes using a Flaming/Brown micropipette puller (P‐97, Sutter Instrument). The extracellular recording solution contained (in mm): 150 NaCl, 5 KCl, 2.5 CaCl_2_, 1 MgCl_2_, 10 glucose, and 10 HEPES (pH 7.4, adjusted with NaOH). The internal solution contained (in mm): 140 KCl, 1 MgCl_2_, 0.5 CaCl_2_, 5 EGTA, 10 HEPES, and 3 Mg‐ATP (pH 7.4, adjusted with KOH). In current‐clamp mode, action potentials were evoked by a 500‐ms depolarizing current injection through the recording electrode. Recordings targeted small‐diameter (Cm < 42 pF) and WGA^+^ neurons, which predominantly consisted of peripheral incision site‐innervating nociceptors. All electrophysiological data were analyzed offline using Clampfit 10.2 software (Molecular Devices, USA).

### 2.13 RNA‐Seq and Data Processing

Tissue from incised plantar hindpaw was harvested for transcriptomic analysis at designated post‐incision time points. The tissues from the surgical site were collected, minced, and immediately stabilized in RNAlater (Thermo Fisher Scientific). Total RNA was then extracted using Trizol reagent (Thermo Fisher Scientific, USA). RNA quality was assessed with TapeStation system (Agilent, USA) and a NanoDrop spectrophotometer (Thermo Fisher Scientific, USA). Only samples meeting stringent quality thresholds: RNA Integrity Number (RIN) ≥ 8.0 and A260/230 ratio ≥ 1.5, were selected for later sequencing. Library preparation and 150 bp paired‐end sequencing were performed on the BGISEQ‐500 platform (BGI Group, Shenzhen, China). For bioinformatics analysis, raw sequencing reads were first aligned to the mouse mm10 reference genome using STAR (v2.5). The resulting alignments were converted from BAM to BED format using BEDTools (v2.17.0). A gene‐count matrix was subsequently generated from the aligned reads with the Rsubread package (v1.30.9). Differential gene expression analysis was performed in R using the DESeq package. Genes with an adjusted *p*‐value (False Discovery Rate, FDR) < 0.001 and an absolute log_2_ fold change > 1.0 were classified as differentially expressed. Functional enrichment and pathway analysis of these gene sets were conducted using the ClusterProfiler package in R.

### 2.14 Histology

Skin tissue samples were collected from mice at postoperative time points of 5 or 7 days. After harvest, the samples were immediately fixed in 10% neutral buffered formalin, processed, and embedded in paraffin for sectioning. To assess tissue morphology and collagen deposition during wound healing, histological sections were stained with Masson's trichrome according to standard protocols. These stained sections were then visualized and imaged under a bright‐field light microscope. For morphometric quantification of wound closure, the histological images were analyzed using ImageJ software.

### 2.15 Western Blot

Five weeks after AAV injection, the animals were euthanized. Following transcardial perfusion with ice‐cold saline, the ipsilateral L3–L5 dorsal root ganglia (DRG) were rapidly dissected on ice, flash‐frozen in liquid nitrogen, and stored at −80°C. Tissues were homogenized in lysis buffer (50 mm Tris, 150 mm NaCl, 0.2% Triton X‐100, pH 7.4) containing protease and phosphatase inhibitor cocktails. The homogenates were centrifuged at 15 000 × g for 15 min at 4°C, and the supernatant was collected for protein concentration determination using a bicinchoninic acid (BCA) assay kit (Thermo Fisher Scientific). Equal protein amounts (20 µg per lane) were separated by SDS‐PAGE on 8% or 15% gels and transferred to polyvinylidene difluoride (PVDF) membranes using a semi‐dry transfer system (Bio‐Rad). Membranes were blocked with 5% bovine serum albumin (BSA) in TBST for 1 h at room temperature before overnight incubation at 4°C with primary antibodies: anti‐CXCR2 (1:1000, #ER1906‐87, Huabio, China) and anti‐TRPA1 (1:1000, #ACC‐037, Alomone, Israel). After washing, membranes were incubated with appropriate horseradish peroxidase‐conjugated secondary antibodies for 1 h at room temperature. Mouse anti–β‐actin (1:5000, #M1210‐5, Huabio, China) served as the loading control. Protein bands were visualized using an enhanced chemiluminescence substrate (BioSharp) and imaged with a FluorChem R system (Bio‐Techne). Densitometric analysis was performed using ImageJ, with target protein expression normalized to β‐actin.

### 2.16 Co‐Immunoprecipitation

Ipsilateral L3–L5 DRGs were rapidly dissected from euthanized mice and homogenized on ice in RIPA lysis buffer (Beyotime, China) containing a protease inhibitor cocktail. After centrifugation at 12 000 × g for 10 min at 4°C, the homogenates were clarified. For co‐immunoprecipitation, the protein lysates were incubated overnight at 4°C with 1 µg of a CXCR2‐targeting primary antibody (GeneTex, USA). Immune complexes were captured by a subsequent 2‐h incubation with protein A/G magnetic beads at 4°C. The beads were washed three times with ice‐cold lysis buffer, and bound proteins were eluted by boiling in loading buffer at 100°C for 10 min. The eluted immunoprecipitates and corresponding input lysates were then separated by SDS‐PAGE and transferred to membranes for standard Western blot analysis.

### 2.17 AAV‐PHP.S‐Mediated Cxcr2 Gene Knockdown

To achieve neuro‐specific knockdown of *Cxcr2* in surgical site‐innervating DRG neurons, we used a recombinant adeno‐associated virus serotype PHP.S (rAAV‐PHP.S) for retrograde transduction. The vector (rAAV‐PHP.S‐hSyn‐shCxcr2‐EGFP, titer: 1.3 × 10^1^ ^3^ vg/mL), constructed by BrainVTA (China), expresses a short hairpin RNA (shRNA) targeting *Cxcr2* mRNA under the human synapsin (hSyn) promoter and includes an EGFP reporter. The specificity and efficiency of AAV‐PHP.S has been validated in our recent studies [6, 27]. We delivered 10 µL of the viral suspension via injection into the deep tissue of the right hindpaw. This injection was performed 5 weeks before the plantar incision surgery to allow time for retrograde transport to the corresponding dorsal root ganglia (DRG) and for maximal transgene expression. Control mice received an equivalent dose of a control vector expressing a scrambled, non‐targeting shRNA sequence (rAAV‐PHP.S‐hSyn‐scramble‐EGFP) under the same conditions. This control accounts for any non‐specific effects from viral transduction and shRNA expression.

### 2.18 Retrograde Labeling of DRG Neurons

For retrograde labeling of DRG neurons, wheat germ agglutinin (WGA)‐594 was injected into the hindpaw site where the incision is to be made 3 days ahead (8.3 mg/mL, 5 µL injection volume). Incision site‐innervating DRG neurons were identified by WGA‐positive staining.

### 2.19 Drug Treatment

CXCL5 neutralizing antibody (3 µg/site; MAB433, R&D Systems, USA) was administered by intraplantar injection into the dorsal part of ipsilateral hindpaw at 1 h before and 5, 23, 47, 71 h after skin incision. Normal goat IgG (isotype control) was dissolved in sterile PBS and given in the same volume and schedule. TRPA1 antagonist HC030031 (10 µg/site) and/or TRPV1 antagonist AMG9810 (67 ng/site) were injected into the dorsal part of mouse hindpaw. 1% DMSO in PBS was used as vehicle control treatment. For pharmacologically blocking CXCR2 downstream pathways in cultured DRG neurons, PI3K inhibitor wortmannin (WMN, 50 nm), MEK1/2 inhibitor U‐0126 (10 µm), STAT3 inhibitor Stattic (10 µm), p38 MAPK inhibitor SB203580 (SB203, 10 µm), CXCR2 antagonist SB225002 (SB225, 100 nm) were applied. Working concentrations for above reagents were adopted from earlier published studies [10, 28, 29, 30, 31].

### 2.20 Ca^2+^ Imaging

Ca^2+^ imaging was performed on dissociated mouse DRG neurons 4–6 h after plating. Neurons were loaded with 10 µm Fura‐2 AM (Thermo Fisher Scientific, USA) in a loading buffer (containing in mm: 140 NaCl, 5 KCl, 2 CaCl_2_, 2 MgCl_2_, and 10 HEPES, pH 7.4) at 37°C for 45 min in the dark. Following incubation, cells were washed three times with the loading buffer and rested for 30 min in the dark prior to imaging as previously described [32, 33]. Ratiometric imaging was conducted using a Nikon ECLIPSE Ti‐S microscope equipped with a Polychrome V monochromator (Till Photonics, USA) and an Orca Flash 4.0 CCD camera (Hamamatsu, Japan). Images were acquired and processed with MetaFluor software (Molecular Devices, USA). The fluorescence ratio (F340/F380) was used to indicate changes in intracellular Ca^2+^ concentration, with exposure times of 0.5 ms at 340 nm and 0.3 ms at 380 nm. Representative pseudocolor images were generated using ImageJ. Data were obtained from 20 to 60 neurons per coverslip. A neuron was considered responsive if its peak Ca^2+^ response exceeded 20% above the baseline, as defined in our previous studies [34].

### 2.21 Immunocytochemistry

Mouse DRG neurons were cultured on poly‐D‐lysine/laminin‐coated coverslips. Immunocytochemistry was performed 4–6 h after plating. The culture medium was aspirated and cells were gently rinsed once with pre‐warmed (37°C) PBS. Neurons were fixed with freshly prepared 4% paraformaldehyde (in 0.01 M PBS) for 20 min at room temperature, followed by three 5‐min washes with PBS. Cells were permeabilized with 0.5% Triton X‐100 in PBS for 15 min at room temperature and then blocked with 5% normal donkey serum for 30 min. They were incubated overnight at 4°C with the primary antibody, rabbit anti‐TRPV1 (1:500, #ACC‐030, Alomone Labs, Israel), rabbit anti‐CXCR2 (1:1000, #GTX14935, Genetex, USA) diluted in the blocking solution. The following day, cells were washed three times with PBS (5 min each) and incubated with appropriate fluorophore‐conjugated secondary antibodies for 1 h at room temperature in the dark. Images were acquired using an Axio Imager M2 microscope (Zeiss, Germany).

### 2.22 Immunostaining

After deep anesthesia with isoflurane, mice were transcardially perfused with 0.9% saline followed by freshly prepared 4% paraformaldehyde (in 0.01 M PBS). The ipsilateral hindpaw skin, L3–5 DRGs, and spleen were then dissected and post‐fixed in the same fixative for 4–6 h at 4°C. Subsequently, tissues were cryoprotected by sequential immersion in 15% and 30% sucrose solutions until they sank. Following embedding in OCT compound, serial frozen sections were cut on a cryostat (NX50, Thermo Fisher Scientific, USA) at thicknesses of 12 µm (skin and spleen) and 8 µm (DRG). Sections were mounted onto gelatin‐coated glass slides. For immunofluorescence staining, sections were blocked with 5% donkey serum in PBS for 1 h at 37°C, followed by overnight incubation at 4°C with the following primary antibodies: rabbit anti‐CXCR2 (1:1000, #GTX14935, Genetex, USA), rat anti‐Ly6G (1:500, #551461, BD Biosciences, USA), rabbit anti NF200 (1:200, #55453S, CST, USA), rabbit anti TRPV1 (1:500, #ACC‐030, Alomone labs, Israel) and rabbit anti‐Iba1 (1:500, #ab178846, Abcam, UK). After six 10‐min washes with PBS, sections were incubated with appropriate fluorophore‐conjugated secondary antibodies for 1 h at 37°C in the dark. Images were acquired using an Axio Imager M2 microscope (Zeiss, Germany).

### 2.23 Statistical Analysis

Data are presented as mean ± SEM. Statistical analyses were performed using GraphPad Prism (8.0). Normality was assessed with Shapiro–Wilk test, and homogeneity of variances was verified using Levene's test before parametric analyses. For comparisons between two groups, parametric data were analyzed with unpaired two‐tailed Student's *t*‐test, while non‐parametric data were evaluated with Mann–Whitney U test. Comparisons involving three or more groups employed one‐way or two‐way ANOVA, as appropriate, with Tukey's post hoc test for parametric data; non‐parametric multi‐group comparisons used Kruskal–Wallis test followed by Dunn's test. *p* < 0.05 was considered statistically significant.

## 3 Results

### 3.1 CXCL5 is a Highly Upregulated Chemokine in the Incised Tissue of a Mouse Model of Skin Plus Deep Tissue Incision and Contributes to Incisional Pain

A mouse model of skin plus deep tissue incision was established to mimic incisional (INC) pain (Figure 1A) [6]. Compared with control mice, INC group mice displayed obvious mechanical allodynia by von Frey test (Figure 1B). Mechanical allodynia lasted over 7 days after incision (Figure 1B). INC group mice also showed obvious heat hyperalgesia that persisted for 5 days (Figure 1C). To explore potential inflammatory mediators or signaling pathways involved in INC pain, we performed RNA‐Sequencing (RNA‐Seq) with tissues (containing both incised skin and muscle) collected from incised area of INC group mice on days 1 and 3 and compared with tissues from control mice (Figure 1D). Figure 1E illustrates the differentially expressed genes (DEGs) identified from INC vs. control group on days 1 and 3. 1626 up‐ and 1963 down‐regulated DEGs were identified from day 1, whereas 1909 up‐ and 1215 down‐regulated DEGs were identified from day 3. We were interested in DEGs that were both upregulated through day 1 and 3. So, we crossed these two datasets and obtained a core set of 1307 DEGs concurrently upregulated through days 1–3 (Figure 1F,G).

**FIGURE 1:**
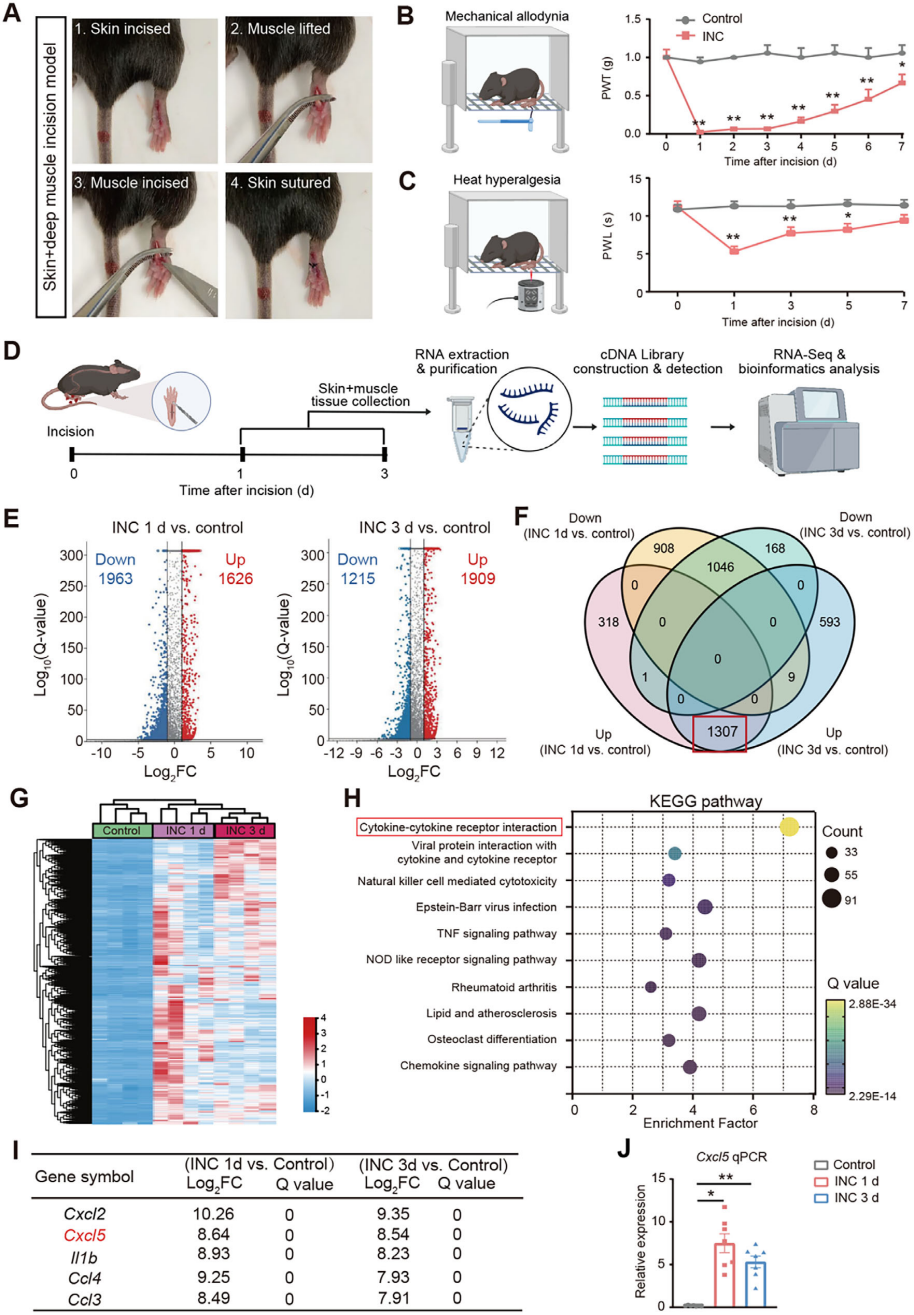
Identification of inflammatory cytokines contributing to INC pain in skin plus deep tissue incision via RNA‐Seq. (A) Model establishment. (B) Paw withdraw threshold (PWT) in hind paw from control vs. INC group mice. *n* = 6 mice/group. (C) Paw withdraw latency (PWL) in hind paw from control vs. INC group mice. (D) Protocol for collecting hind paw tissues from incision site for bulk RNA‐Seq. (E) Volcano plot showing DEGs identified from INC 1 days vs. control and INC 3 days vs. control group. *n* = 4 mice/group. (F) Venn diagram showing overlapped DEGs from 1 and 3 days. (G) Heat map showing the core set of 1307 DEGs that were both upregulated at 1 and 3 days time points. (H) KEGG analysis of this core set of DEGs. (I) List of top 5 most upregulated DEGs in cytokine‐cytokine receptor interaction as in panel H. Please note all measured Q‐values were close to zero, and are therefore presented as 0 in the table. (J) qPCR validation of *Cxcl5* gene. One‐way ANOVA with Tukey's post hoc test for panel J.

KEGG analysis of these DEGs identified a series of enriched signaling pathways, including cytokine‐cytokine receptor interaction, viral protein interaction with cytokine, etc. (Figure 1H). We were interested in cytokine‐cytokine receptor interaction since this pathway may include genes involved in pain and tissue inflammation. We examined the top‐ranking DEGs. *Cxcl5*, a gene implicated in pain modulation [10, 35], stood out as one of the most significantly upregulated genes (Figure 1I). qPCR confirmed *Cxcl5* was upregulated in incised tissues of INC model mice through days 1–3 (Figure 1J).

Since the incised tissues contained both skin and muscle, we wanted to know the specific tissue origin where *Cxcl5* was upregulated. qPCR showed *Cxcl5* gene was significantly upregulated in both skin and muscle tissues of INC group through days 1–5. ELISA confirmed CXCL5 protein expression was increased in both skin and muscle tissues of INC model mice (Figure 2A,B). We further checked CXCL5 expression in human skin wound. We reanalyzed a single‐cell RNA‐Seq dataset of human skin wound healing [36]. Bioinformatics indicated an increase of human *CXCL5* gene expression especially in myeloid cells and fibroblasts cluster in human skin wound edge vs. intact skin through days 1–7 after skin wound creation (Figure S1).

**FIGURE 2:**
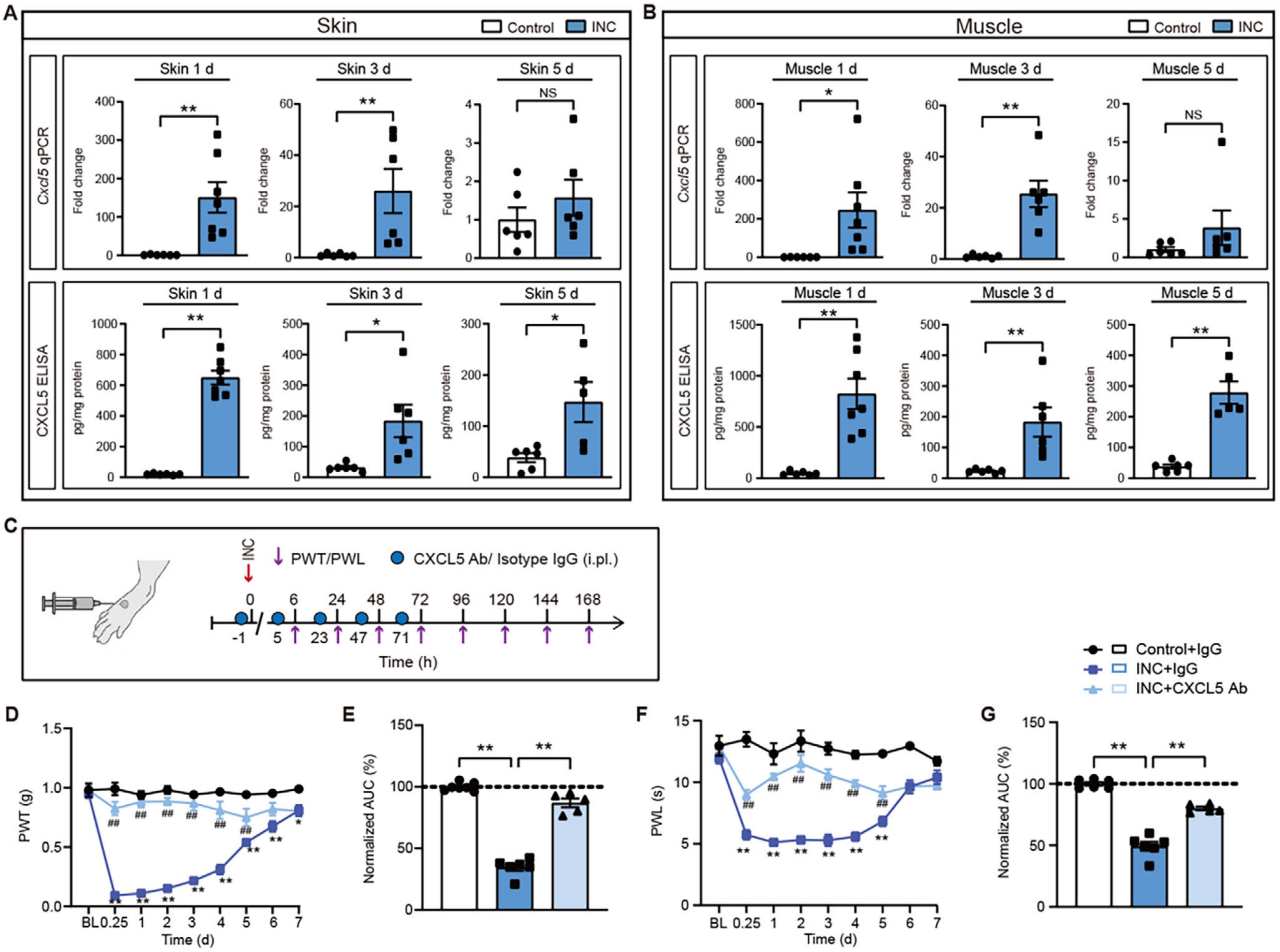
CXCL5 is upregulated in the incised site and contributes to INC pain. (A) *Cxcl5* gene and CXCL5 protein expression in skin tissues through days 1–5. (B) *Cxcl5* gene and CXCL5 protein expression in muscle tissues through days 1–5. (C) Schematic picture showing experiment schedule. CXCL5 neutralizing antibody or isotype control IgG was injected into the dorsal part of the ipsilateral hindpaw at time points as shown. (D) PWT changes in Control+IgG, INC+IgG, and INC+CXCL5 Ab groups. ^*^ *p* < 0.05, ^**^ *p* < 0.01 vs. Control+IgG group. ^##^ *p* < 0.01, ^#^ *p* < 0.05 vs. INC+IgG group. (E) Normalized area under the curve (AUC) analysis of traces as in panel D. (F) PWL changes in mice. (G) AUC analysis of traces as in panel F. *n* = 5–7 mice/group. ^**^ *p* < 0.01, ^*^ *p* < 0.05. Unpaired Student's *t*‐test for panel A&B. Two‐way ANOVA with Tukey's post hoc test for panel D&F. One‐way ANOVA with Tukey's post hoc test for panel E&G.

We examined the role of CXCL5 in INC pain model mice. We injected a monoclonal neutralizing antibody against CXCL5 (3 µg/site) or isotype control IgG into the dorsal part of the hindpaw at −1, 5, 23, 47, and 71 h time points as shown in Figure 2C. CXCL5 neutralizing antibody significantly ameliorated both mechanical allodynia and heat hyperalgesia of INC model mice vs. isotype control IgG (Figure 2D–G). Furthermore, immunostaining revealed that CXCL5 neutralizing antibody reduced neutrophil recruitment in skin tissues near the incisional site (Figure S2A,B). These results show that CXCL5 is a highly upregulated chemokine in the incised tissues of a mouse model of INC pain and contributes to mechanical and heat pain hypersensitivity as well as neutrophil recruitment.

### 3.2 CXCL5 Signals Through Neuronal CXCR2 to Contribute to INC Pain, Gait Impairment, and Emotional Disorder

Our recent work indicates that CXCL5 can activate CXCR2 expressed in sensory neurons to produce pain in a mouse model of acute gouty arthritis [10]. We then postulate that the increased CXCL5 in incised tissues might activate neuronal CXCR2 to produce INC pain. The expression of CXCR2 in DRG neurons was further confirmed by immunocytochemistry on cultured DRG neurons. We observed strong CXCR2 expression in DRG neurons, including on cell membrane (Figure S3A,B). Publicly available single‐cell RNA‐seq datasets (GSE255436) from mouse DRG also demonstrates that *Cxcr2* gene is expressed in DRG neurons (Figure S4) [37, 38]. We then generated *Cxcr2* global knockout (*Cxcr2* gKO) mice by deleting *Cxcr2* coding region in Exon 2 via Cas9/gRNA technique (Figure 3A). We found that *Cxcr2* gKO mice showed significantly reduced mechanical allodynia vs. wild type (WT) mice after INC through days 1–6 (Figure 3B). Area under the curve (AUC) analysis indicated ameliorative effect of *Cxcr2* gKO on mechanical allodynia of INC pain model mice throughout observation frame (Figure 3C). Although *Cxcr2* gKO improved mechanical allodynia, it came to our mind that *Cxcr2* gKO potentially delays wound healing [39]. We indeed observed a significant delay in wound healing process in *Cxcr2* gKO mice compared with WT mice when excisional wound was created in nape of neck (Figure 3D,E). Histopathology with Masson staining further indicated delayed wound healing occurred in *Cxcr2* gKO mice (Figure 3F,G). Thus, although *Cxcr2* gKO strategy effectively ameliorates INC pain, it possesses an obvious drawback in causing wound healing delay.

**FIGURE 3:**
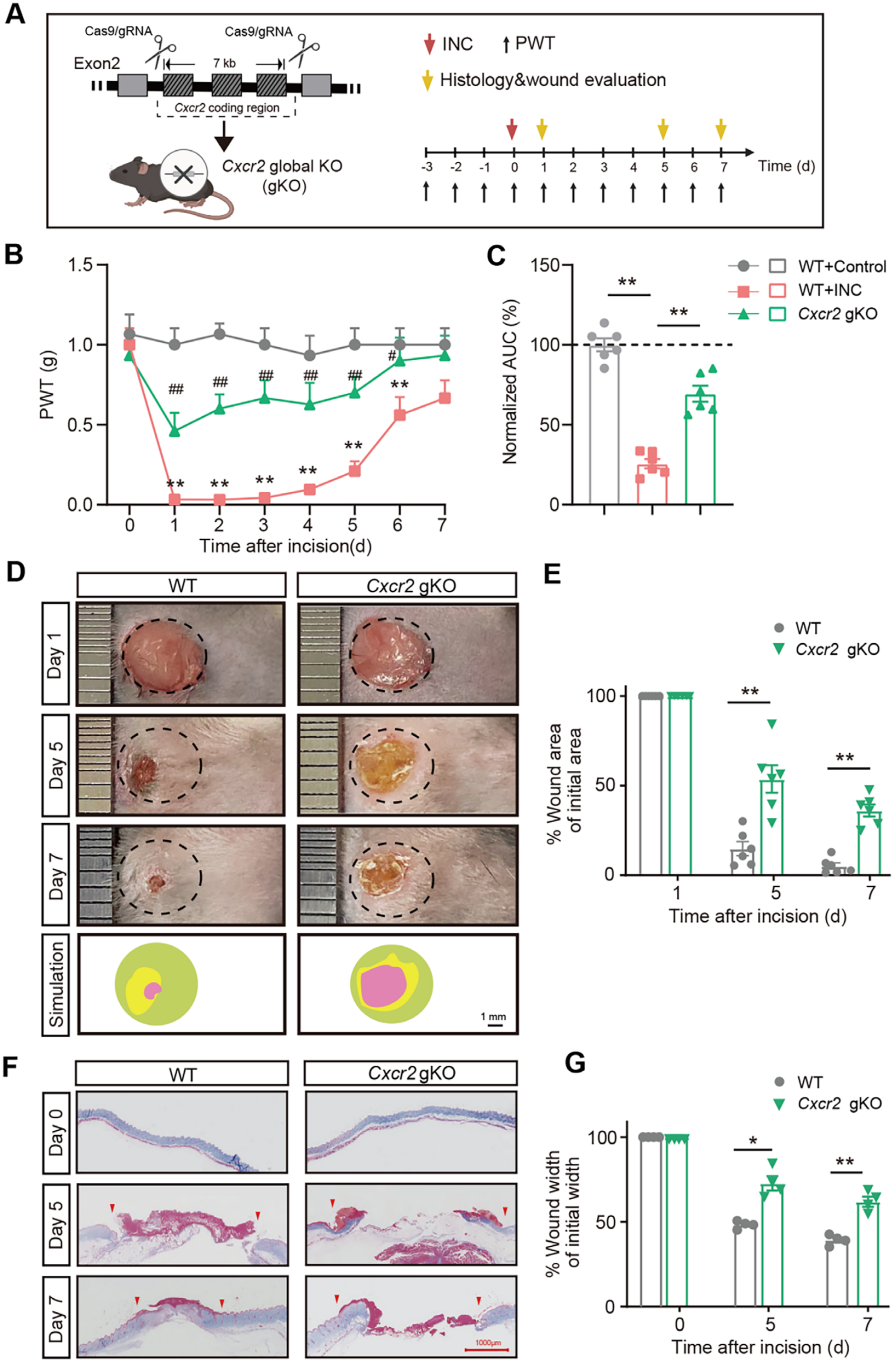
*Cxcr2* gKO ameliorates INC pain but causes significant delay in wound healing. (A) Strategy of generating *Cxcr2* gKO mouse strain via Cas9/gRNA. (B) PWT in hind paw from WT+Control, WT+INC and *Cxcr2* gKO+INC groups. ^**^ *p* < 0.01 vs. WT+Control group. ^##^ *p* < 0.01 and ^#^ *p* < 0.05 vs. WT+INC group. (C) AUC analysis of curves shown in panel B. Values from WT+Control group was taken as 100%. *n* = 6 mice/group. (D) Wound healing in WT and *Cxcr2* gKO mice upon a circular incision (5 mm in diameter) in nape of neck. Lower panels show simulated skin wound on days 1, 5, and 7 overlaid for comparison. (E) Summary of % of wounded area/initial area in WT and *Cxcr2* gKO mice from panel D. *n* = 6 mice/group. (F) Masson staining showing skin histology before and after incision (days 5 and 7). Red arrowhead indicates wounded area. (G) Summary of % of wounded area/initial area in WT and *Cxcr2* gKO mice from panel F. *n* = 4 mice/group. ^**^ *p* < 0.01, ^*^ *p* < 0.05. Two‐way ANOVA with Tukey's post hoc test for panel B, E&J. One‐way ANOVA with Tukey's post hoc test for panel C.

We then generated a mouse line with conditional deletion of *Cxcr2* (*Cxcr2* cKO) in peripheral nociceptive neurons expressing *Nav1.8* with the aid of SNS‐Cre (Figure 4A) [40]. *Cxcr2* cKO mice showed significantly reduced CXCR2 expression in DRG neurons (Figure 4B,C). For comparison, CXCR2 expression was completely ablated in DRG neurons isolated from *Cxcr2* gKO mice (Figure 4B,C). In contrast, CXCR2 expression in the spleen and splenic neutrophils remained unchanged in *Cxcr2* cKO mice (Figure 4D,E). Collectively, these findings validated the specificity of the CXCR2 antibody used herein and verified the efficacy of our conditional knockout approach targeting peripheral sensory neurons. Next, we examined the wound healing in *Cxcr2* cKO mice. No abnormality in wound healing was observed in *Cxcr2* cKO mice (Figure 4F–H). Histopathology reveals similar wound healing process in *Cxcr2* cKO mice vs. *Cxcr2* ^fl/fl^ mice (Figure 4I,J). We examined the nocifensive behavior of *Cxcr2* cKO mice when INC pain model was established. *Cxcr2* cKO mice showed significantly improved paw withdrawal threshold (PWT) as well as paw withdrawal frequency (PWF) stimulated by von Frey hair vs. *Cxcr2* ^fl/fl^ mice (Figure 4K–P). *Cxcr2* cKO mice displayed improved heat hyperalgesia vs. *Cxcr2* ^fl/fl^ mice (Figure 4Q,R). These results indicate CXCL5 signals through neuronal CXCR2 to mediate INC pain and *Cxcr2* cKO is a superior strategy to ameliorate INC pain without affecting wound healing.

**FIGURE 4:**
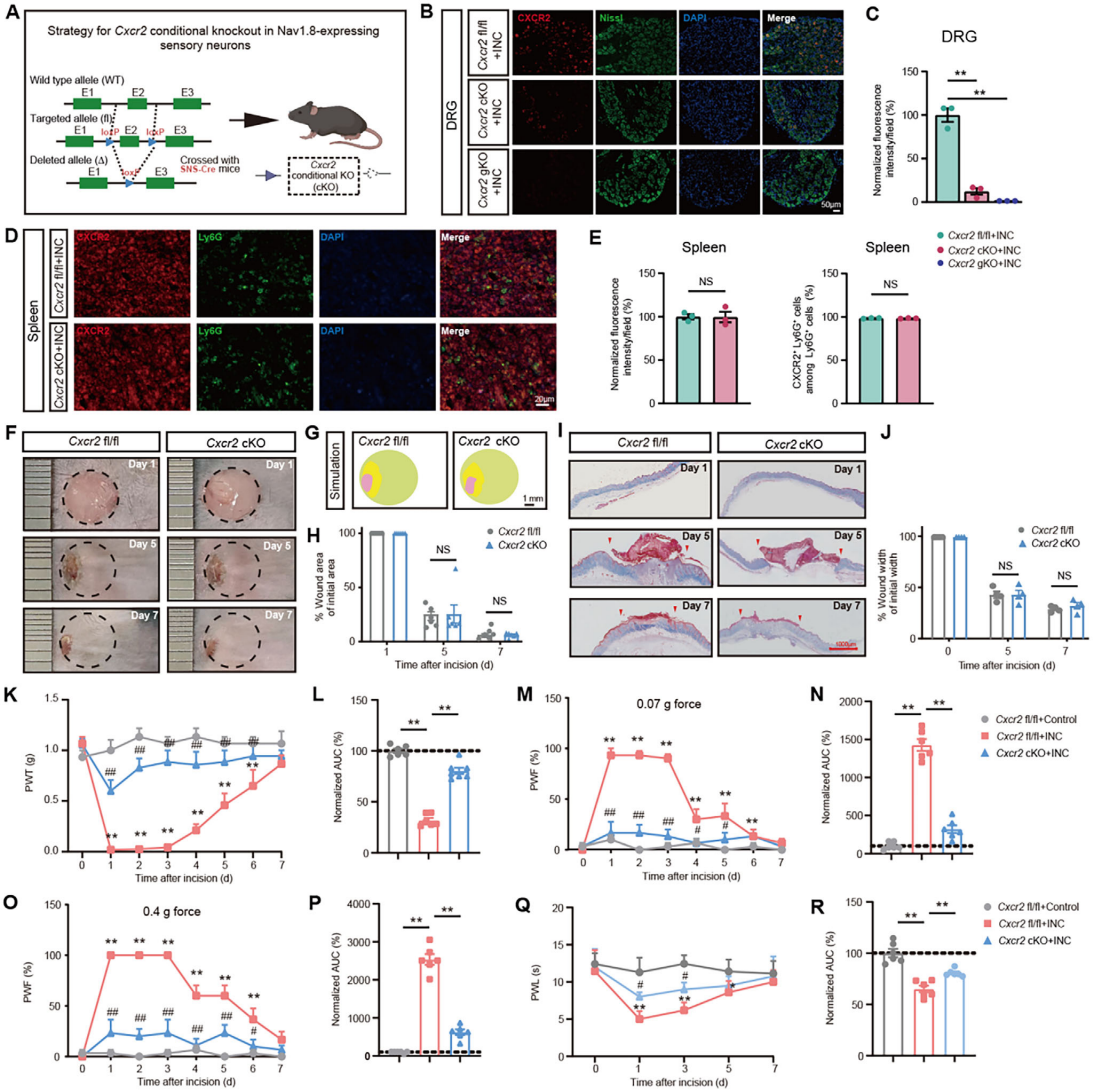
*Cxcr2* cKO ameliorates INC pain without affecting wound healing. (A) Strategy of generating *Cxcr2* cKO mouse strain in Nav1.8‐expressing sensory neurons. (B) Immunostaining showing CXCR2 expression in DRG neurons from *Cxcr2* ^fl/fl^, *Cxcr2* cKO, and *Cxcr2* gKO mice. (C) Summary of normalized fluorescence intensity of CXCR2 in DRG of three groups. *n* = 3 mice/group. (D) Immunostaining showing CXCR2 expression in the spleen. (E) Left: summary of normalized fluorescence intensity of CXCR2 in spleen. Right: summary of CXCR2^+^Ly6G^+^ cells among Ly6G^+^ cells in spleen. (F) Wound healing in *Cxcr2* ^fl/fl^ and *Cxcr2* cKO mice upon circular incision. (G) Overlaid wound simulations. (H) Summary of % of wounded area/initial area in *Cxcr2* ^fl/fl^ and *Cxcr2* cKO mice. *n* = 6 mice/group. (I) Masson staining showing skin histology before and after incision (day 5 and 7) in *Cxcr2* ^fl/fl^ and *Cxcr2* cKO mice. Red arrowhead indicates wounded area. (J) Summary of % of wounded area/initial area in *Cxcr2* ^fl/fl^ and *Cxcr2* cKO mice. *n* = 4 mice/group. (K) PWT in ipsilateral hind paws of mice from days 0 to 7. ^**^ *p* < 0.01 vs. *Cxcr2* ^fl/fl^+Control group. ^##^ *p* < 0.01 and ^#^ *p* < 0.05 vs. *Cxcr2* ^fl/fl^+INC group. (L) Normalized AUC analysis of curves in panel K. (M) Paw withdraw frequency (PWF) upon 0.07 g force stimulation in ipsilateral hind paws through days 0–7. (N) AUC analysis of curves in panel M. (O) PWF changes upon 0.4 g force stimulation. (P) AUC analysis of curves in panel O. (Q) PWL in ipsilateral hind paws. (R) AUC analysis of curves in panel Q. *n* = 6–7 mice/group. ^**^ *p* < 0.01. NS: no significance. Two‐way ANOVA with Tukey's post hoc test for panel H, J, K, M, O, and Q. One‐way ANOVA with Tukey's post hoc test for panel L, N, P, and R. Unpaired Student's *t* test for panel C&E.

INC pain intensifies during movement, restricting patient's ability to move and perform daily activities. Our prior study showed that INC pain model mice displayed significant pain‐related gait impairment [6]. Therefore, we examined whether *Cxcr2* cKO may help improve gait impairment. Gait analysis indicated that *Cxcr2* ^fl/fl^+INC group mice showed obvious impairment in gait parameters vs. *Cxcr2* ^fl/fl^+control group on days 1 and 3 after INC. *Cxcr2* cKO+INC group showed significantly improved gait parameters vs. *Cxcr2* ^fl/fl^+INC group on days 1 and 3 (Figure S5A–C,E,F). We employed a more detailed spatiotemporal analysis to characterize dynamic changes in footprints. Our findings indicated *Cxcr2* ^fl/fl^+INC group exhibited abnormal dynamic alteration in the area of affected hind paw vs. *Cxcr2* ^fl/fl^+control group on days 1 and 3, which was reversed in *Cxcr2* cKO+INC group mice (Figure S5D,G).

The above tests were done in female mice. We further tested if neuronal CXCR2 signaling contributes to INC pain in male mice. In male mice, we found *Cxcr2* cKO also significantly alleviated INC‐induced mechanical allodynia and heat hyperalgesia, a result consistent with female mice (Figure S6A,D). These data indicates CXCR2 in nociceptive sensory neurons is responsible for INC pain of both sexes.

People with postsurgical pain develop emotional disorders, e.g. anxiety and depression, which correlate with pain severity and are critical determinants for overall pain experience [41, 42]. We explored whether INC pain model mice showed signs of emotional disorders. In elevated plus maze (EPM) test, *Cxcr2* ^fl/fl^+INC group mice spent less time in the open arm compared with *Cxcr2* ^fl/fl^+control group, which was reversed in *Cxcr2* cKO+INC group mice (Figure S7A–C). In open field test, *Cxcr2* ^fl/fl^+INC group mice spent less time in the central area of the open field vs. *Cxcr2* ^fl/fl^+control group mice. This aberrant behavior was reversed in *Cxcr2* cKO+INC group mice (Figure S7D–F). All groups of mice traveled similar distances in total in both open field and EPM tests. These results demonstrate that neuronal CXCR2 contributes to gait impairment and anxiety‐related behavior of INC pain model mice.

### 3.3 Neuronal CXCR2 Expression and Its Coupling With TRPA1 Channel are Enhanced in INC Pain Condition

We next focused on DRG neurons specifically innervating the incised tissue since these neurons are potentially involved in pain signal generation during INC pain. To identify peripheral sensory neurons that innervate the incised tissue, we employed retrograde labelling strategy using wheat germ agglutinin (WGA). WGA‐594 was injected into the location of hind paw where incision is to be made 3 days ahead of incision/sham operation (Figure 5A). Ipsilateral L3‐L5 DRG were harvested (Figure 5A). Immunostaining revealed that the expression of CXCR2 was significantly increased in INC model group than control group (Figure 5B,C). We next specifically assessed CXCR2^+^WGA^+^ DRG neurons and found that their percentage among Nissl^+^ DRG neurons was likewise significantly elevated in INC group (Figure 5B,D). Our recent work revealed a coupling exists between CXCR2 and TRPA1 in sensory neurons that CXCR2 activation by CXCL5 signals through TRPA1 to induce neuronal hyperexcitability and generate pain signal [10]. Since CXCR2 expression was upregulated in DRG neurons, we wanted to know whether its coupling with TRPA1 was also enhanced in INC pain condition. Co‐immunoprecipitation (Co‐IP) using anti‐CXCR2 antibody showed the existence of a coupling between TRPA1 and CXCR2 in DRG of control mice, a result consistent with our prior study (Figure 5E) [10]. We observed more CXCR2 expression in total protein input for Co‐IP in INC model group vs. control group, whereas TRPA1 expression was unchanged (Figure 5F,G). Importantly, the CXCR2‐TRPA1 coupling was significantly enhanced in DRG from INC model mice (Figure 5H).

**FIGURE 5:**
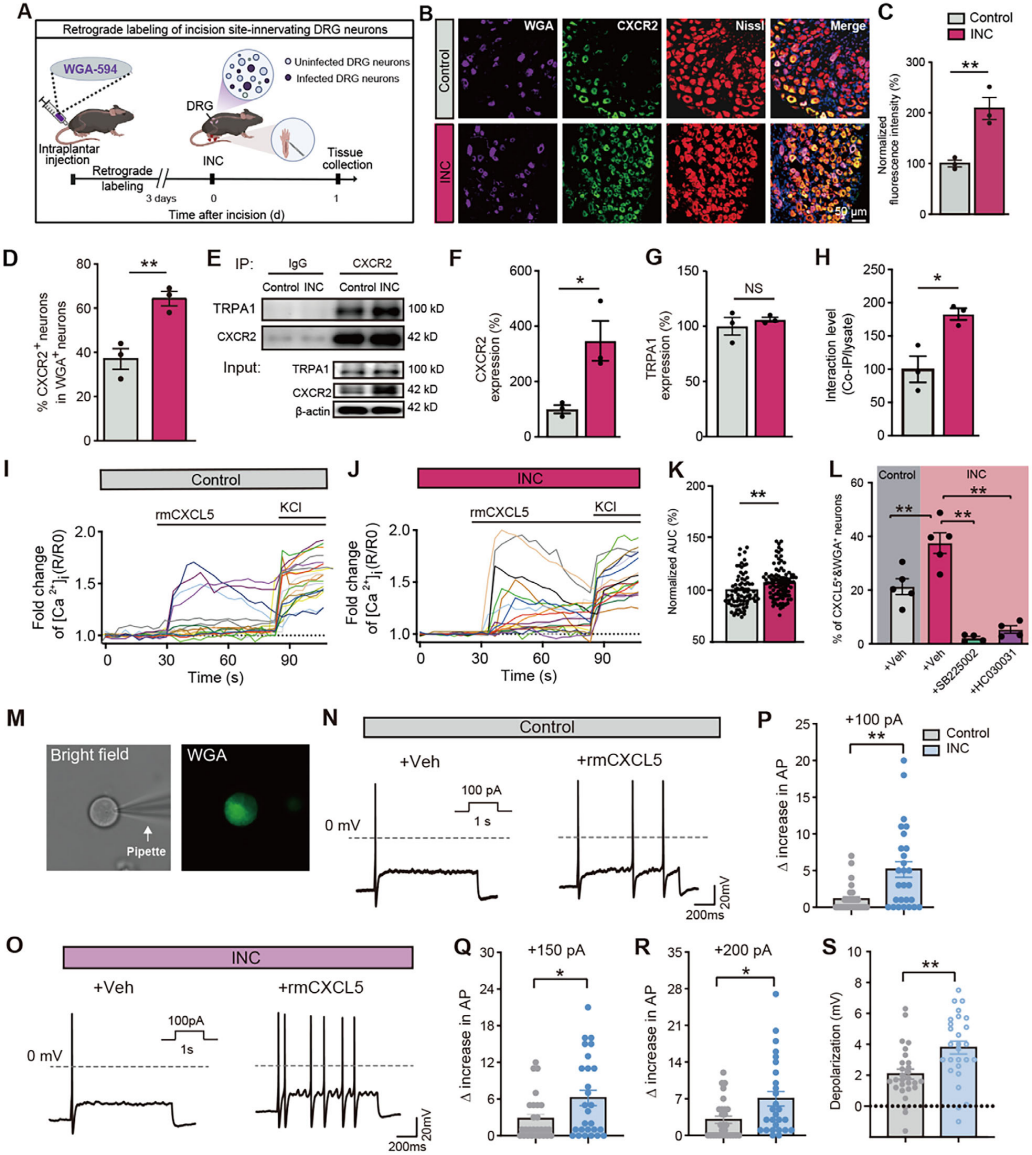
CXCR2 expression and its coupling with TRPA1 channel are enhanced in DRG neurons during INC pain. (A) Retrograde labeling of incision site‐innervating DRG neurons using WGA‐594. (B) Immunostaining showing CXCR2 expression in incision site‐innervating DRG neurons labeled by WGA. (C) Summary of total CXCR2 expression in DRG of control and INC model mice. (D) Summary of % CXCR2^+^ neurons among WGA^+^ neurons. (E) Co‐IP showing coupling of CXCR2 with TRPA1 in DRG lysates from control and INC groups. (F&G) Summary of CXCR2 and TRPA1 expression in DRG lysates. (H) CXCR2‐TRPA1 interactional level by Co‐IP. *n* = 3 tests/group. (I&J) Ca^2+^ traces in WGA^+^DRG neurons from control and INC group. 20 WGA^+^ neurons were included in each group. (K) Area under the curve analysis (AUC) of CXCL5‐induced Ca^2+^ responses in WGA^+^ DRG neurons from control and INC group. 98 and 106 neurons were included, respectively. (L) Summary of % of CXCL5^+^ WGA^+^ neurons among KCl^+^ neurons. SB225002 (100 nm) and HC030031 (100 µm) were used to block CXCR2 and TRPA1. *n* = 4–5 tests/group. Each test includes 40–60 neurons/field. (M) Selection of a WGA^+^DRG neurons under the microscope with WGA‐594 fluorescence. (N&O) Current clamp recording of WGA^+^DRG neurons from control or INC group in the presence of vehicle (Veh, 0.1% BSA) or after rmCXCL5 application. Dashed line: 0 mV. 100 pA current was injected to evoke APs. (P–R) Summary of Δ increase in the number of APs induced by rmCXCL5 with 100, 150, or 200 pA current injection in WGA^+^DRG neurons. (S) Membrane depolarization induced by rmCXCL5 of WGA^+^DRG neurons. *n* = 28 and 27 neurons for control and INC group, respectively. ^**^ *p* < 0.01, ^*^ *p* < 0.05. One‐way ANOVA with Tukey's post hoc test for panel L. Unpaired Student's *t* test for others.

To determine whether the enhanced CXCR2‐TRPA1 coupling may result in functional changes, we performed ratiometric Ca^2+^ imaging in DRG neurons from control and INC model mice that were pre‐labeled with WGA (Figure 5A). WGA^+^ neurons were picked for analysis for Ca^2+^ responses specifically in incised tissue‐innervating DRG neurons. Our results indicated recombinant mouse CXCL5 (rmCXCL5, 100 nm) elicited stronger response in WGA^+^ DRG neurons from INC group than from control group (Figure 5I–K). Besides, the % of WGA^+^ DRG neurons showing response to rmCXCL5 was also increased in INC model group than control (Figure 5L). Ca^2+^ imaging showed that a majority of CXCL5‐responding DRG neurons also respond to the TRPA1 specific agonist AITC (100 µm) (Figure S8A,B). Blocking CXCR2 or TRPA1 with specific antagonist SB225002 or HC030031 almost completely blocked rmCXCL5‐induced Ca^2+^ response in INC model group (Figure 5L), demonstrating that the enhanced Ca^2+^ response was completely due to enhanced CXCR2‐TRPA1 coupling in DRG neurons.

We then wanted to know whether the functionally enhanced CXCR2‐TRPA1 coupling eventually contributed to DRG neuron hyperexcitability. We specifically selected WGA^+^DRG neurons with small sizes (Cm < 42 pF) and performed patch clamp recording (Figure 5M) since these small‐sized WGA^+^ neurons are predominantly nociceptive neurons and innervate the incised site. Excitability was induced under current clamp mode by injecting 100, 150, and 200 pA current. rmCXCL5 application elicited more action potentials (APs) in WGA^+^DRG neurons from INC pain model group than control (Figure 5N–R). Moreover, rmCXCL5 produced stronger membrane potential depolarization of WGA^+^DRG neurons from INC pain model group vs. control group (Figure 5S). These results indicate neuronal CXCR2 expression and its coupling with TRPA1 are enhanced in INC pain, resulting in DRG neuron hyperexcitability upon CXCR2 activation by CXCL5.

### 3.4 CXCL5 Enhances TRPV1 Channel Activity via CXCR2‐Mediated Signaling in DRG Neurons

Since *Cxcr2* cKO mice displayed significantly reduced heat hyperalgesia in INC pain condition, we wondered whether CXCL5‐CXCR2 signaling might modulate the heat‐sensitive TRPV1 channel. We checked whether CXCL5 affects TRPV1 expression in DRG neuron culture. qPCR showed rmCXCL5 (20 or 100 nm) incubation for 2 h upregulated *Trpv1* expression in DRG neuron culture (Figure 6A,B). Immunocytochemistry confirmed that TRPV1 protein expression was significantly upregulated in cultured DRG neurons (Nissl^+^) by rmCXCL5 incubation (20 or 100 nm, Figure 6C,D). We performed Ca^2+^ imaging on cultured DRG neurons to examine whether TRPV1 functional activity was upregulated after CXCL5 treatment. TRPV1 channel was activated by applying capsaicin (100 nm). We found CXCL5 (20 nm) incubation significantly promoted % of DRG neurons responding to capsaicin (Figure 6E–H). CXCR2 is coupled with several downstream signaling, including p38 MAPK, STAT3, and PI3K, etc. (Figure 6I). Pharmacological blockage of CXCR2 or p38 MAPK significantly reduced CXCL5‐induced potentiation of TRPV1 activity in DRG neurons, whereas blocking other pathways did not affect the effect of CXCL5 (Figure 6J).

**FIGURE 6:**
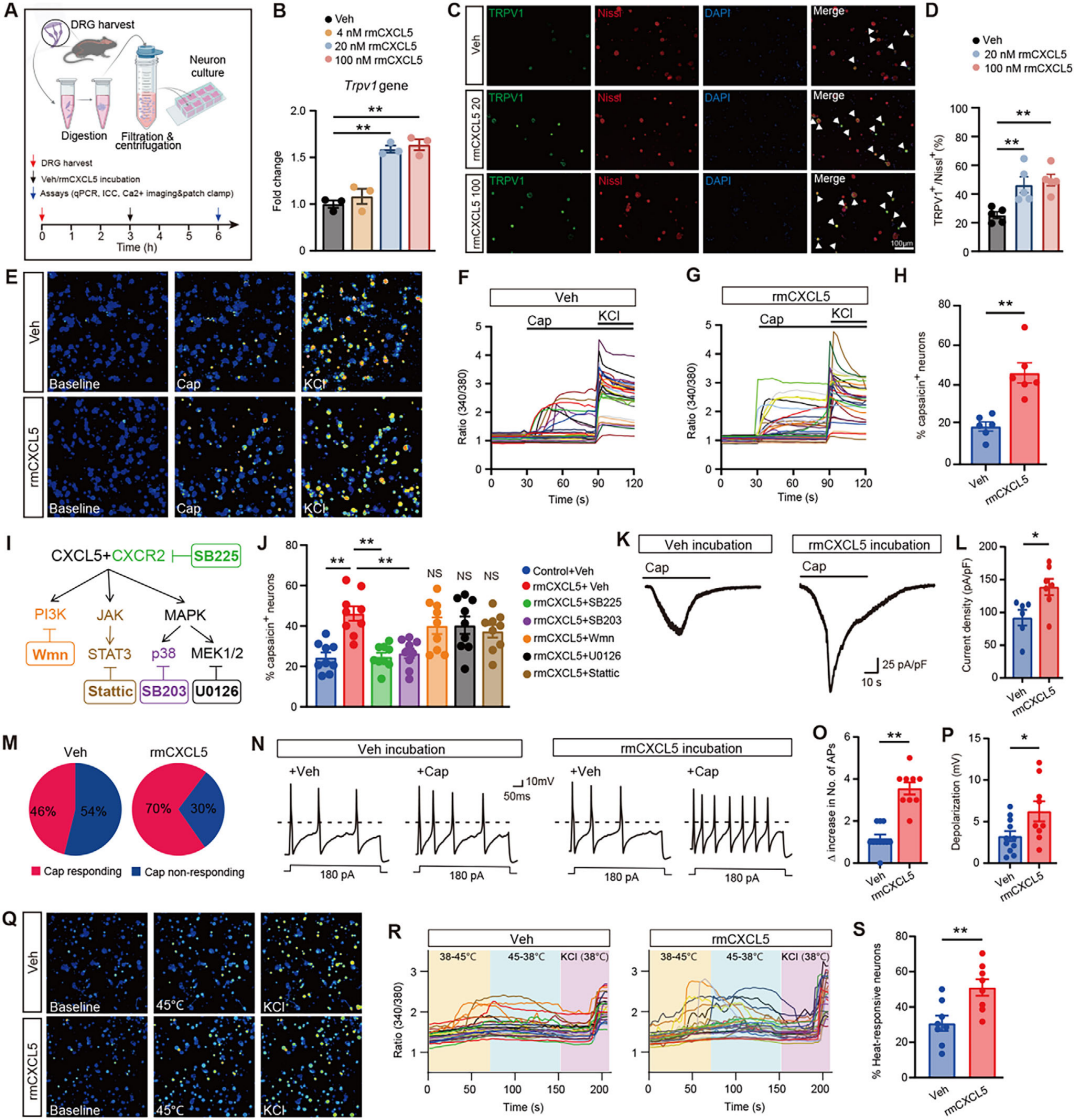
CXCL5 promotes TRPV1 channel functional expression via CXCR2‐mediated intracellular signaling in DRG neurons. (A) Experimental scheme. (B) qPCR showing *Trpv1* expression in DRG neuron culture treated with vehicle (Veh, 0.1% BSA) or rmCXCL5 (4, 20, or 100 nm). (C) ICC of TRPV1 expression in DRG neurons. (D) Summary of TRPV1^+^ neurons in Nissl^+^ DRG neurons after rmCXCL5 treatment. (E) Ca^2+^ transients triggered by capsaicin (100 nm) in DRG neurons after vehicle or rmCXCL5 (20 nm) incubation. (F&G) Overlaid Ca^2+^ traces in DRG neurons treated with vehicle or rmCXCL5. 30 neurons were included per group. (H) Summary of % of capsaicin^+^KCl^+^ neurons among KCl^+^ neuron. *n* = 6 tests/group. (I) Pharmacological treatments against CXCR2‐related downstream signaling. (J) Summary of % of capsaicin^+^KCl^+^ neurons among KCl^+^ neuron after pharmacological treatments. *n* = 9 tests/group. (K) Representative TRPV1 channel currents elicited by capsaicin in Veh‐ or rmCXCL5‐incubated DRG neurons. (L) Summary of TRPV1 current density. *n* = 6–7 neurons/group. (M) Pie charts showing % of capsaicin‐responding neurons. (N) Representative APs triggered by applying capsaicin (100 nm) in Veh‐ or rmCXCL5‐incubated groups under current clamp recording. (O) Summary of Δ increase in the No. of APs triggered by capsaicin. (P) Membrane potential depolarization induced by capsaicin in DRG neurons. *n* = 11 and 9 neurons for Veh‐ and rmCXCL5‐incubated group, respectively. (Q) Representative Ca^2+^ imaging showing Ca^2+^ responses triggered by 45°C noxious heat stimulation in DRG neurons with vehicle or rmCXCL5 incubation. (R) Overlaid Ca^2+^ traces in DRG neurons of two groups. 30 neurons were included per group. (S) Summary of % of noxious heat‐responsive DRG neurons. *n* = 8 tests/group. One‐way ANOVA with Tukey's post hoc test for panel B, D&J. Unpaired Student's *t*‐test for others.

Whole‐cell patch clamp further confirmed that rmCXCL5 incubation promoted TRPV1 channel current density and increased % of capsaicin‐responding neurons in cultured DRG neurons (Figure 6K–M). We tested whether CXCR2 signaling‐induced TRPV1 upregulation could result in DRG neuron hyperexcitability. Current clamp was used to record DRG neuron excitability. Capsaicin application triggered more APs in nociceptive DRG neurons from rmCXCL5‐treated group than vehicle‐treated group (Figure 6N,O). Moreover, capsaicin triggered more membrane depolarization in DRG neurons from rmCXCL5‐treated group vs. vehicle‐treated group (Figure 6P). Since TRPV1 is well known as a sensor for noxious heat, we tested whether CXCL5 incubation might enhance noxious heat‐stimulated TRPV1 activity in DRG neurons. Ca^2+^ imaging indicated that rmCXCL5 incubation significantly increased percentage of DRG neurons responding to noxious heat stimulation (45°C) (Figure 6Q–S).

Prior work demonstrates functional up‐regulation of TRPV1 in DRG neurons contributes to incisional pain. However, the exact mechanisms underlying how TRPV1 is upregulated remain elusive [43]. We tested whether neuronal CXCR2 contributes to TRPV1 upregulation in DRG neurons in INC pain model mice. Ca^2+^ imaging combined with WGA retrograde labeling showed that TRPV1 channel activity was upregulated in DRG neurons innervating the incised site in *Cxcr2* ^fl/fl^+INC mice vs. *Cxcr2* ^fl/fl^+control mice 2 days after the incision. The upregulated TRPV1 channel activity was reduced in DRG neurons innervating the incised site in *Cxcr2* cKO+INC mice (Figure 7A–C). We proceeded to test the involvement of p‐38 MAPK in TRPV1 overexpression in DRG neurons and heat hyperalgesia in INC model mice (Figure S9A). Immunostaining showed that there was more TRPV1 expression in DRG neurons innervating the incisional site (as indicated by WGA^+^ staining) from INC group than from control group mice 2 days after the incision was made (Figure S9B,C). Pharmacological blocking p‐38 MAPK by SB203580 (10 nmol/mouse, intrathecal administration) significantly reduced TRPV1 overexpression in WGA^+^ DRG neurons of INC model mice (Figure S9B,C). Behavioral assay further validated that blocking p‐38 MAPK activity improved heat hyperalgesia of INC model mice (Figure S9D). Therefore, these results indicate that CXCL5 potentiates TRPV1 channel activity via neuronal CXCR2‐dependent p‐38 MAPK signaling in DRG neurons of INC pain model mice.

**FIGURE 7:**
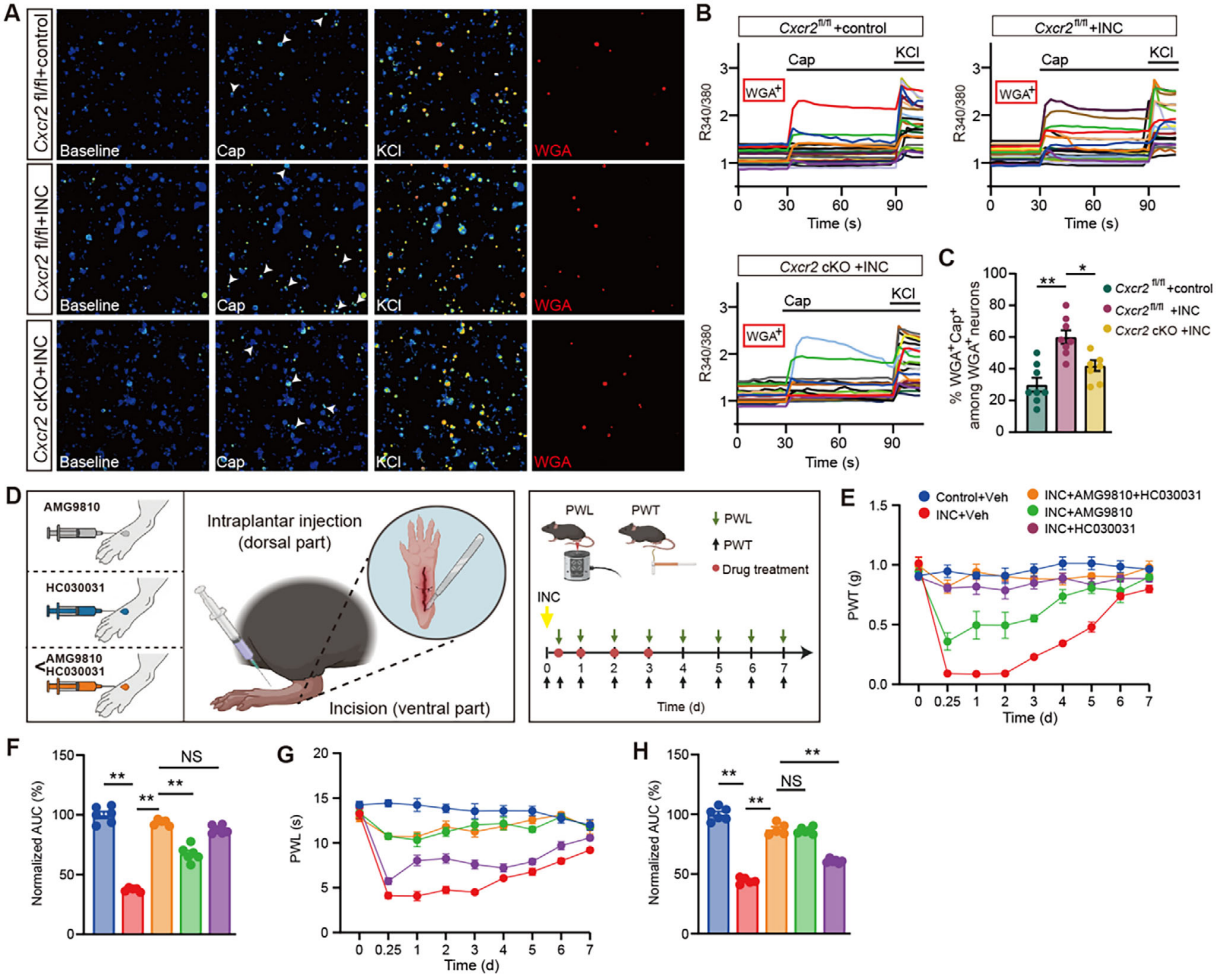
Neuronal CXCR2‐dependent upregulation of TRPV1 expression in DRG neurons innervating incision site and sufficient pain relief via synergistic TRPA1 and TRPV1 blockade. (A) Ca^2+^ imaging of DRG neurons derived from *Cxcr2* ^fl/fl^+control, *Cxcr2* ^fl/fl^+INC, and *Cxcr2* cKO+INC groups of mice. WGA was used to label incision site‐innervating DRG neurons. White arrows indicate WGA^+^Capsaicin^+^KCl^+^ neurons. (B) Overlaid Ca^2+^ traces of WGA^+^DRG neurons upon capsaicin (100 nm) and KCl (40 mm) challenge from three groups. 25 WGA^+^DRG neurons were included per group. (C) % of WGA^+^Capsaicin^+^ neurons among WGA^+^DRGneurons of three groups. *n* = 7–8 fields derived from three mice/group. (D) Cartoon showing pharmacological blockade of TRPA1 and/or TRPV1 and experimental schedule. (E) PWT changes. (F) AUC analysis of curves in panel E. (G) PWL changes. (H) AUC analysis of curves in panel G. *n* = 5–6 mice/group. ^**^ *p* < 0.01, ^*^ *p* < 0.05. One‐way ANOVA with Tukey's post hoc test for panel C, F, and H.

Since neuronal CXCR2 modulates both TRPA1 and TRPV1, we wanted to know whether TRPA1 and TRPV1 concurrently mediated INC pain. TRPA1 or TRPV1 channel was pharmacologically blocked by intraplantar injection of specific antagonist HC‐030031 or AMG9810 (Figure 7D). Mechanical allodynia was largely reversed by TRPA1 blockade alone but only partially by TRPV1 blockade. Heat hyperalgesia was largely reversed by TRPV1 blockade alone but only partially by TRPA1 blockade. The combined blockade of both channels offers more effective alleviation of both mechanical and heat pain hypersensitivities in INC pain model mice (Figure 7E–H).

### 3.5 *Cxcr2* cKO in Nociceptive Sensory Neurons Does not Affect Local Inflammatory Response in Incision Site

Inflammation triggers and potentiates incisional pain. But inflammation per se constitutes a critical component for wound healing that contributes to the protection and repair of damaged tissues [44]. We tested whether *Cxcr2* cKO might affect inflammation in incisional site. To identify potential inflammation processes that might be affected, we screened through RNA‐Seq using tissues from *Cxcr2* ^fl/fl^+control, *Cxcr2* ^fl/fl^+INC, and *Cxcr2* cKO+INC group (Figure S10A). DEGs were identified by volcano plot and heat map (Figure S10B–D). KEGG analysis of these DEGs revealed that the top enriched pathways were not obviously different between *Cxcr2* ^fl/fl^+INC and *Cxcr2* cKO+INC groups (Figure S10E).

We examined expression of some typical genes involved in inflammation by RNA‐Seq and found these genes were all upregulated to a similar extent in incised tissues of *Cxcr2* cKO group compared with *Cxcr2* ^fl/fl^ group (Figure S10F). qPCR showed that some typical pro‐inflammatory genes were all similarly upregulated in the incised tissues of *Cxcr2* cKO group compared with *Cxcr2* ^fl/fl^ group (Figure S10G). ELISA showed expression of CGRP and substance P were similarly upregulated in incised tissues of *Cxcr2* cKO group vs. *Cxcr2* ^fl/fl^ group (Figure S10H). We next monitored macrophages and neutrophils, two groups of inflammatory cells important for tissue inflammation and wound healing, in the incised site. Immunostaining showed that the immunostaining intensity of Iba1 and Ly6G were similarly increased in both *Cxcr2* ^fl/fl^+INC group and *Cxcr2* cKO+INC group vs. control on days 1 and 3 after incision (Figure S10I–K). These results demonstrate that *Cxcr2* cKO in nociceptive sensory neurons does not affect local inflammatory response in the incision site.

### 3.6 Targeted *Cxcr2* Knockdown in Incision Site‐Innervating DRG Neurons Reduces INC Pain

We tested whether targeted knockdown of *Cxcr2* in incision site‐innervating DRG neurons could produce similar therapeutic effect on INC pain as *Cxcr2* cKO. We adopted AAV‐PHP.S that was developed as a capsid subtype specifically for peripheral neuron transfection and possess retrograde infection property [6, 45, 46]. The neuronal promoter hSyn was further adopted to guide specific neuronal expression. AAV‐PHP.S‐hSyn‐shRNA*Cxcr2*‐EGFP or scrambled control was administered to hind paw where incision was to be made. Then incision was made 5 weeks after the transfection (Figure 8A). We confirmed viral expression by immunostaining. There was strong EGFP signal in ipsilateral but not in contralateral L3‐L5 DRG neurons of mice being transfected (Figure 8B). We performed immunostainings to characterize these EGFP^+^ neurons. We found that around 49.4% EGFP‐positive DRG neurons co‐express with TRPV1, whereas 22.7% EGFP‐positive DRG neurons co‐express with NF‐200 (Figure S11A,B). These results indicate that EGFP^+^ DRG neurons include both small to medium‐sized nociceptors (TRPV1^+^) and large myelinated Aβ fiber neurons (NF200^+^). We then performed cell size analysis of EGFP^+^ DRG neurons and all DRG neurons (Nissl^+^). The result showed that cell size distribution patterns of EGFP‐positive DRG neurons encompass small, medium to large sizes, which is similar with the patterns derived from all DRG neurons (Figure S11C). Therefore, the EGFP^+^ DRG neurons transfected by AAV PHP.S‐hSyn include both nociceptors and mechanoreceptors.

**FIGURE 8:**
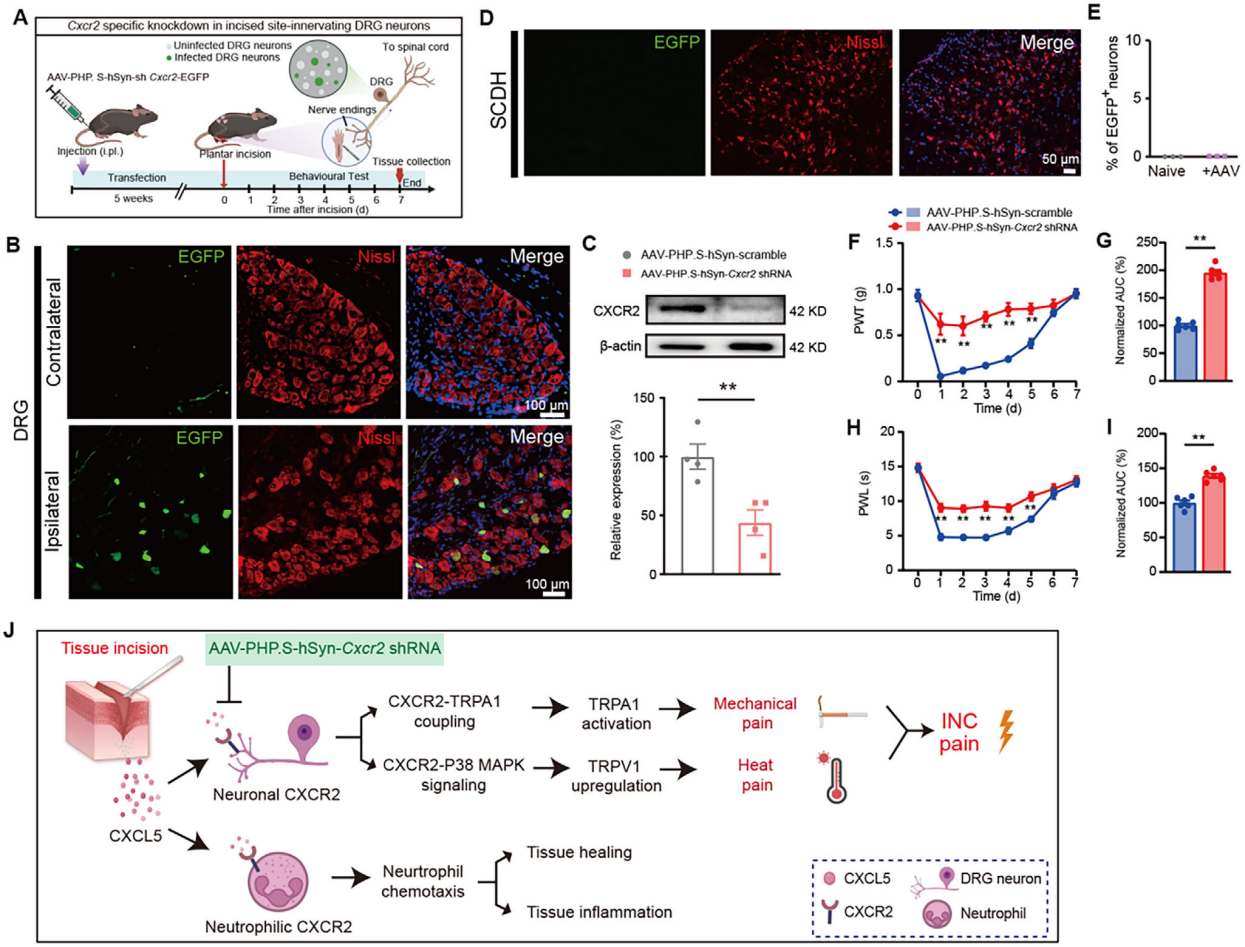
Targeted knockdown of *Cxcr2* expression in incision site‐innervating DRG neurons effectively reduces INC pain. (A) Strategy for *Cxcr2*‐specific knockdown in incised site‐innervating DRG neurons using AAV‐PHP.S. AAV‐PHP.S‐hSyn‐sh *Cxcr2*‐EGFP or AAV‐PHP.S‐hSyn‐sh scramble‐EGFP was injected into the site where incision is to be made 5 weeks ahead of incision. (B) Immunostaining showing EGFP expression in ipsilateral and contralateral L3‐L5 DRG of transfected mice. (C) Western blot examining CXCR2 expression in ipsilateral L3‐L5 DRG of mice being transfected. *n* = 4 mice/group. (D) EGFP signal detection in spinal cord dorsal horn (SCDH) of mice being transfected. (E) Summary of % of EGFP^+^ SCDH neurons in naïve mice and mice being transfected. *n* = 3 mice/group. (F) Time course showing PWT changes. (G) Normalized AUC analysis of curves as shown in panel F. (H) Time course showing PWL changes. (I) Normalized AUC analysis of curves as shown in panel H. *n* = 6 mice/group. (J) Working model for targeting neuronal CXCR2 for INC pain management without affecting tissue healing and inflammation. Two‐way ANOVA with Tukey's post hoc test was used for panel F&H. Student's *t‐*test (unpaired) was used for panel C, E, G, and I.

Western blot further verified CXCR2 protein expression was decreased in ipsilateral L3‐L5 DRG of mice transfected with AAV‐PHP.S‐hSyn‐sh*Cxcr2*‐EGFP vs. control vector (Figure 8C). Spinal cord dorsal horn (SCDH) showed no detectible EGFP signals (Figure 8D,E). These results confirmed the specificity and efficiency of our strategy to knockdown *Cxcr2* specifically in incision site‐innervating DRG neurons. We established INC pain model in these two groups of mice. Targeted *Cxcr2* knockdown in incision site‐innervating DRG neurons ameliorated both mechanical allodynia and heat hyperalgesia in hind paw of INC pain model mice (Figure 8F–I). These results demonstrate that targeted knockdown of *Cxcr2* in incision site‐innervating DRG neurons effectively relieves incisional pain.

## 4 Discussion

We demonstrated a critical role of CXCL5‐neuronal CXCR2 signaling in nociceptive sensory neurons that mediates INC pain via concurrently activating TRPA1 and sensitizing TRPV1. By analyzing human single‐cell RNA‐Seq dataset, we further found human *CXCL5* is also upregulated in human skin wound tissues. Our work reveals some major findings as below: 1) CXCL5 is upregulated in both incised skin and muscle tissues and contributes to INC pain. 2) *Cxcr2* gKO improved INC pain but caused significant delay in wound healing, whereas *Cxcr2* cKO in nociceptive sensory neurons improved INC pain without affecting wound healing and tissue inflammation. 3) In DRG neurons innervating the incision, CXCR2 expression and coupling with TRPA1 were enhanced, causing hyperexcitability upon CXCL5 stimulation. CXCL5 further potentiated TRPV1 via CXCR2‐mediated p38 MAPK signaling. 4) The modulation of TRPA1/TRPV1 by CXCL5‐CXCR2 signaling in DRG neurons concurrently promotes mechanical and heat pain hypersensitivities in INC pain.

CXCL5 can be produced by macrophages, mast cells, fibroblasts, etc. upon stress or inflammation [47, 48, 49]. These cells can be activated upon skin or tissue incision [6, 50, 51]. In this study, we reanalyzed a single‐cell RNA‐Seq dataset of human skin wound healing and bioinformatics indicated an increase of human CXCL5 gene expression especially in myeloid cells and fibroblasts cluster in human skin wound edge vs. intact skin through days 1–7 after skin wound creation. Therefore, we speculate that CXCL5 can be produced via several potential cellular sources, including macrophages, mast cells, and fibroblasts upon tissue incision. Further studies would be needed to further clarify the cellular expression of CXCL5 upon skin plus muscle incision.

CXCR2 is expressed in nociceptive sensory neurons [10, 11]. Our recent work revealed that CXCR2 is functionally coupled with TRPA1 channel in DRG neurons [10]. Our work further demonstrate CXCL5 can promote DRG neuron hyperexcitability via CXCR2‐ and TRPA1‐dependent mechanism [10]. After tissue incision, CXCL5, released from incised skin and muscle, acts upon CXCR2 expressed in nociceptive sensory neurons to activate TRPA1, resulting in sensory neuron excitability and pain signal generation. Here we demonstrate that CXCR2 expression is significantly upregulated in sensory neurons innervating the incised tissues. This increased CXCR2 expression can enhance CXCR2‐TRPA1 coupling in DRG neurons from INC pain model mice. We verified that CXCL5 induced stronger TRPA1 channel activation in DRG neurons innervating the incised tissue, resulting in hyperexcitability of peripheral sensory neurons. These results indicate that neuronal CXCR2 expression and its coupling with TRPA1 channel are enhanced under INC pain. This mechanism likely contributes to peripheral sensitization during INC pain, a process known to amplify pain signals at the site of injury and facilitate pain transmission. Therefore, the enhanced CXCR2‐TRPA1 signaling axis may represent a key mechanism underlying the transition from acute to persistent pain after tissue incision.

According to our RNA‐Seq analysis, *Cxcl2* gene expression is also highly upregulated. Nevertheless, our prior work demonstrated that CXCL2 acts as a much weaker agonist for neuronal CXCR2 relative to CXCL5. In that study, we performed Ca^2+^ imaging to compare the stimulatory potency of CXCL1, CXCL2, and CXCL5 in primary cultured DRG neurons [10]. CXCL5 elicited the most potent neuronal activation among three chemokines, whereas CXCL1 exhibited weaker activity than CXCL5. In contrast, CXCL2 triggered minimum stimulatory responses. These functional data indicate that CXCL1 and CXCL5 serve as potent agonists for neuronal CXCR2, whereas CXCL2 only weakly engages this receptor [10]. We further analyzed *Cxcl1* gene expression in the incised tissues from our RNA‐seq dataset and confirmed its marked upregulation (with 2^5.9 fold increase on days 1 and 2^4.5 fold increase on day 3) during INC pain. We therefore propose that CXCL1 and CXCL5 secreted from incised tissue are major chemokines that activate CXCR2 on nociceptive neurons. This signaling cascade subsequently triggers TRPA1 activation and elevates TRPV1 expression. The combined actions of these two chemokines may jointly drive INC pain.

Our study further identifies TRPV1 as another downstream target of neuronal CXCR2. In contrast to TRPA1, CXCR2 activation by CXCL5 does not directly produce TRPV1 channel activation [10]. But here we found CXCR2 activation could significantly potentiate TRPV1 channel activity. We further found CXCL5 promoted an increase in TRPV1 channel expression via CXCR2‐dependent mechanism in DRG neurons. This result indicates that the potentiating effect on TRPV1 was likely due to increased channel expression. CXCR2 is associated with several downstream signaling, including p38 MAPK, STAT3, and PI3K, etc. [52]. Among these signaling, p38 MAPK and PI3K have been reported to promote TRPV1 expression and functional activity in sensory neurons [53, 54]. We found that blocking p38 MAPK, but not other signaling, significantly reduced CXCR2‐mediated potentiating effect on TRPV1. We further found TRPV1 channel activity was significantly enhanced in DRG neurons innervating incised site of INC pain model mice. The enhanced TRPV1 activity was attenuated in *Cxcr2* cKO mice. These results indicate that CXCL5 potentiates TRPV1 channel activity via CXCR2‐mediated intracellular signaling in DRG neurons of INC pain model mice. These results further demonstrate that neuronal CXCR2‐induced potentiating effect on TRPV1 in DRG neurons contributes to peripheral sensitization during INC pain. Collectively, these findings highlight a critical role of neuronal CXCR2 as a functional modulator of nociceptor excitability during INC pain, which acts in concert with TRPA1 and TRPV1 to maintain a hyperexcitable state of DRG neurons.

Our findings also highlight the distinct yet complementary roles of TRPA1 and TRPV1 channels in pathogenesis of incisional pain. While both channels are known to be critical detectors of noxious stimuli, our results suggest that their contributions to INC pain hypersensitivity are mechanistically divergent. Previous studies have shown that TRPV1 knockout mice exhibited reduced heat hypersensitivity but unaltered mechanical hypersensitivity after skin incision [55]. It is demonstrated that TRPA1 is involved in mechanical allodynia but not heat hyperalgesia of INC pain model mice [6, 56]. Moreover, in a mouse model of oral mucosa and whisker pad skin incision model, it is demonstrated that combined pharmacological blockade of TRPV1 and TRPA1 produces superior analgesic effect on both mechanical and heat pain hypersensitivity compared with blockade of either channel alone [57]. Here in our study, we found TRPV1 acts as a primary sensor for heat hyperalgesia, whereas TRPA1 functions more prominently in transducing mechanical allodynia in the skin plus deep tissue incision model mice. Therefore, the coordinated modulation of TRPA1 and TRPV1 by CXCL5‐CXCR2 in DRG neurons creates a synergistic mechanism that amplifies nociceptor excitability and subsequently promotes mechanical and heat pain hypersensitivity in tissue incision.

In our recent work, we found that *Cxcr2* cKO significantly alleviated tissue inflammation, neutrophil infiltration and CGRP/SP production in ankle joint tissues of a mouse model of acute gouty arthritis (AGA) [10, 14]. This observation is in contrast with our current findings derived from INC pain model mice. We speculate this discrepancy could originate from the distinct disease animal models we tested. In AGA model, CXCL5 activates neuronal CXCR2‐TRPA1 signaling to activate DRG neurons and produce joint pain. In the meantime, the activation of DRG neurons triggers the release of neuropeptides CGRP and substance P in the inflamed joint tissues. These two neuropeptides then mediated vasodilation and plasma extravasation that further facilitate CXCL5‐induced neutrophil infiltration in the inflamed joint. Therefore, CXCL5‐neuronal CXCR2‐TRPA1 axis contributes to pain, neutrophil influx, and inflammation in AGA model mice. In contrast, the INC pain model differs from AGA model in that it involves the incision of both skin and the muscle underneath. The incision severs both the blood vessels and the nerves, which per se causes the infiltration of large amounts of neutrophils and release of neuropeptides such as CGRP and substance P [58, 59]. These processes are largely independent of neuronal CXCR2‐TRPA1 signaling. This mechanistic difference may explain why *Cxcr2* cKO exerts no detectable effects on tissue inflammation and neutrophil infiltration in the INC pain model.

CXCR2 has been known as a crucial participant in skin wound healing. Prior studies demonstrated global knockout of CXCR2 causes significant delay in skin wound healing caused by both skin incision and chemical injury [39, 60]. CXCR2 global knockout mice showed significantly reduced immune cell recruitment to wound site [39]. This is likely due to the loss of CXCR2 function on immune cells, particularly neutrophils, which rely on this receptor for chemotaxis to the wound site [7, 39]. A growing body of evidence has confirmed that neutrophils are critically involved in promoting skin wound repair [61, 62]. Clinical studies found that patients with reduced neutrophil levels often suffer from delayed wound healing [63]. Moreover, accumulated neutrophils produce reactive oxygen species, release granular contents, and form NETs to eradicate invading bacteria and pathogens in wound site, a process important for tissue healing [7, 63]. Here we found mice with *Cxcr2* cKO in nociceptive sensory neurons showed normal skin wound healing, a finding drastically different from *Cxcr2* gKO mice. Neutrophil and macrophage infiltrations as well as tissue inflammation in incised site of *Cxcr2* cKO mice were not altered. Our findings that *Cxcr2* gKO and cKO mice exhibit distinct pain and wound healing phenotypes suggests the cellular substrates driving pain and tissue repair are mechanistically separable in INC pain condition, despite their reliance on a common mediator CXCR2. These findings highlight therapeutic potential of developing neuron‐specific *Cxcr2* interference strategies that can effectively dissociate analgesic benefits from detrimental effects on tissue healing by selectively targeting neuronal CXCR2 (Figure 8J).

## 5 Conclusion

Our work reveals a critical role of neuronal CXCR2 signaling in nociceptive sensory neurons mediating INC pain. Targeting sensory neuronal CXCR2 may represent an effective strategy for INC pain relief without affecting wound healing.

## Author Contributions

Boyi L., C.Y., C.W., and J.F. designed research; C.Y., P.L., Y.P., Y.C., Z.D., and Y.L. performed research; Boyu L., Y.L., and Y.T. contributed new reagents/analytic tools; C.Y., P.L., Y.P., Y.C., and Z.D. analyzed data; Boyi L. wrote the paper.

## Funding

This work was supported by the National Natural Science Foundation of China (82474625, 82505747, 82505322, and 82305369), Zhejiang Provincial Natural Science Funds (LZ23H270001& LQN25H270018), National High Level Traditional Chinese Medicine Hospital Clinical Research Funding (DZMG‐XZYY‐23001), and the Key Project of Precision Medicine Joint Fund of Natural Science Foundation of Hebei Province (H2025201097)

## Ethics Statement

Animal experiment procedures were approved by the Animal Ethics Committee of Zhejiang Chinese Medical University (#IACUC‐20201214‐13).

## Conflicts of Interest

The authors declare no conflicts of interest

## Supporting information




**Supporting File**: advs77085‐sup‐0001‐SuppMat.docx.

## Data Availability

The data that support the findings of this study are available from the corresponding author upon reasonable request.
