# Supplementary material for: Targeting CXCR2 in Nociceptive Sensory Neurons Offers Novel Therapeutic Potentials for Postoperative Pain
Source: Adv Sci (Weinh). 2026 Aug 11:e77085. Online ahead of print. doi: 10.1002/advs.77085 (PMC13460279; doi:10.1002/advs.77085)
Supplement: Supplementary file 1 [file ADVS-9999-e77085-s001.docx]

**Supplementary figures and legends**

**Fig. S1 *CXCL5* gene expression in human skin wound.** (A) UMAP plot of cell clusters identified by scRNA-seq in human skin wound tissues. (B) Overlaid expression of human *CXCL5* gene in control, wound 1d&7d among all cell clusters. (C) Distribution of human *CXCL5* gene among cell clusters identified in human skin wound samples. “Control” represent intact skin. Wound 1d&7d represent tissues collected from skin wound edge through Day 1 to 7 after skin wound creation. (D) Summary of the expression of *CXCL5* gene across major cell types in human skin. The dataset was reanalyzed from a published scRNA-seq dataset (GSE241132).

**Fig. S2 CXCL5 neutralizing antibody reduces neutrophil recruitment in skin tissues near incisional site.** (A) Representative immunostaining pictures showing neutrophil (by Ly6G positive staining) infiltration in skin tissues of 3 groups of mice. (B) Summary of the No. of Ly6G^+^ cells/mm^2^ of 3 groups as shown in panel A. n=3 mice/group. ^**^p<0.01. One-way ANOVA with Tukey’s post hoc test was used.

**Fig. S3 CXCR2 expression in cultured DRG neurons. (A)** Representative immunostaining pictures of cultured mouse DRG neurons showing CXCR2 expression (in red). Neurons were labeled with neuronal marker NeuN (in green). DAPI was shown in purple. Neurons were cultured 6 h before immunostaining. (B) Cross-sectional analysis of CXCR2 immunofluorescence intensity of the representative neuron shown in panel A.

**Fig. S4 Mouse *Cxcr2* expression analyzed from a published RNA-Seq dataset.** (A) UMAP plot of cell clusters identified by scRNA-seq in mouse DRG. The dataset was reanalyzed from a published scRNA-seq dataset ([GSE255436](https://www.ncbi.nlm.nih.gov/geo/query/acc.cgi?acc=GSE201654)). (B) Distribution of mouse *Cxcr2* gene among different cell clusters identified in mouse DRG by UMAP plot. (C) Analysis of mouse *Cxcr2* gene expression abundance among different cell clusters in DRG.

**Fig. S5 *Cxcr2* cKO improves gait impairment of INC pain model mice.** (A) Time points for gait analysis. (B) Representative pictures showing footprints of mice by gait analysis 1 day after incision/control operation (Day 1). LF: left forepaw; RF: right forepaw; LH: left hind paw; RH: right hind paw. (C) Summary of normalized stance ratio (RH/LH), stride length ratio (RH/LH) and paw area ratio (RH/ LH) of *Cxcr2*^fl/fl^+control, *Cxcr2*^fl/fl^+INC and *Cxcr2* cKO+INC groups of mice 1 day after incision/control operation. The values of *Cxcr2*^fl/fl^+control were taken as 100% and values from other groups were normalized accordingly. n=7 mice/group. (D) Overlaid dynamic changes in ensemble hind paw area obtained from 3 groups on Day 1. (E) Representative pictures showing the footprints of mice by gait analysis 3 days after incision/control operation (Day 3). (F) Summary of normalized stance ratio (RH/LH), stride length ratio (RH/LH) and paw area ratio (RH/ LH) of 3 groups of mice 3 days after incision/control operation. n=6-7 mice/group. (G) Overlaid dynamic changes in ensemble hind paw area obtained from 3 groups on Day 3. ^**^p<0.01, ^*^p<0.05. Two-way ANOVA with Tukey’s post hoc test was used for panel D&G. One-way ANOVA with Tukey’s post hoc test was used for other panels.

**Fig. S6 *Cxcr2* cKO ameliorates mechanical allodynia and heat hyperalgesia in male INC pain model mice.** (A) Time courses showing 50% PWT changes in *Cxcr2*^fl/fl^+control, *Cxcr2*^fl/fl^+INC and *Cxcr2* cKO+INC group of mice. (B) Normalized AUC analysis of traces as shown in panel A. ^*^p<0.05, ^**^p<0.01 vs. *Cxcr2*^fl/fl^ +control group. ^#^p<0.05, ^##^p<0.01 vs. *Cxcr2*^fl/fl^+INC group. (C) Time courses showing PWL changes in *Cxcr2*^fl/fl^+control, *Cxcr2*^fl/fl^+INC and *Cxcr2* cKO+INC group of mice. (D) Normalized AUC analysis of traces as shown in panel C. n=6-7 mice/group. Two-way ANOVA with Tukey’s post hoc test was used for panel A&C. One-way ANOVA with Tukey’s post hoc test was used for panel B&D.

**Fig. S7 *Cxcr2* cKO improves pain-related anxiety like behavior in INC pain model mice.** (A) Representative photos showing the movement traces of *Cxcr2*^fl/fl^+control, *Cxcr2*^fl/fl^+INC and *Cxcr2* cKO+INC groups of mice during elevated plus maze (EPM) test. (B) Summary of the total distance traveled by 3 groups of mice during EPM test. (C) Summary of the time travelled in open arm of 3 groups of mice. (D) Representative photos showing the movement traces of 3 groups of mice during open field test. (E) Summary of the total distance traveled by 3 groups of mice during open field test. (F) Summary of the time travelled in the center zone of 3 groups of mice. n=11-13 mice/group. ^**^p<0.01, ^*^p<0.05. One-way ANOVA with Tukey’s post hoc test was used for analysis.

**Fig. S8 Ca^2+^ imaging showing the responsiveness of CXCL5^+^ DRG neurons to AITC challenge.** (A) Representative Ca^2+^ responses in DRG neurons challenged by CXCL5 (100 nM), followed with TRPA1 agonist AITC challenge (100 μM). (B) Venn diagram showing the overlapping of CXCL5^+^ neurons with AITC^+^ neurons. A total of 132 neurons (KCl^+^) derived from 3 mice were included for this analysis.

**Fig. S9 p38 MAPK signaling contributes to TRPV1 overexpression in DRG neurons and heat hyperalgesia of INC model mice.** (A) Time schedule for p38 MAPK inhibitor SB203580 and corresponding vehicle administration in mice. SB203580 (10 nmol/mouse) or corresponding vehicle (Veh, 0.1% DMSO in PBS) was administrated via intrathecal (i.t.) injection. (B) Representative immunostaining pictures showing TRPV1 expression in DRG neurons that innervate the incisional site from control+Veh, INC+Veh and INC+SB203580 groups. WGA was used to label the incisional site-innervating DRG neurons. (C) Summary of % of WGA^+^TRPV1^+^ neuron among WGA^+^ neuron of 3 groups. n=3-4 mice/group. (D) Time course showing paw withdrawal latency (PWL) changes in ipsilateral hindpaws of 3 groups of mice. n=6 mice/group. One-way ANOVA with Tukey’s post hoc test was used for panel C analysis. Two-way ANOVA with Tukey’s post hoc test was used for panel D analysis.

**Fig. S10 *Cxcr2* cKO in nociceptive sensory neurons does not affect inflammatory response in the incision site.** (A) Experimental design. (B&C) Volcano plots showing DEGs identified from whole incised tissues of *Cxcr2*^fl/fl^+INC vs. *Cxcr2*^fl/fl^+control and *Cxcr2* cKO+INC vs. *Cxcr2*^fl/fl^+INC group. (D) Heatmap showing DEGs identified from 3 groups. (E) KEGG analysis of DEGs in shown panel D. (F) Summary of expression changes of some typical inflammation-related genes from RNA-Seq. (G) qPCR validation of some typical inflammation-related gene. (H) Substance P (SP) and CGRP expression in the incised tissues of 3 groups. (I-K) Macrophage (Iba-1^+^) or neutrophil (Ly6G^+^) infiltration in skin tissues.

**Fig. S11 Characterization of AAV-PHP.S-hSyn vector-transfected DRG neuron.** (A) Immunostaining showing the co-expression of EGFP with TRPV1 in DRG neurons. Neurons were labeled with Nissl (in purple). DAPI (in blue) was used as counterstain. The dotted box covered area is further enlarged and shown on the right panels. Statistics was illustrated on the right. (B) Immunostaining showing the co-expression of EGFP with NF200 in DRG neurons. Statistics was illustrated on the right. (C) Comparison of cell size distribution between EGFP^+^ Nissl^+^ DRG neurons and Nissl^+^ DRG neurons.
